# Features of soil behavior in the near-fault zones during the 2011 Tohoku mega-thrust earthquake Mw 9

**DOI:** 10.1038/s41598-020-65629-2

**Published:** 2020-05-26

**Authors:** Olga V. Pavlenko

**Affiliations:** Schmidt Institute of Physics of the Earth, Russian Academy of Sciences, B. Gruzinskaya 10, Moscow, 123242 Russia

**Keywords:** Solid Earth sciences, Seismology

## Abstract

Soil behavior is studied during the Tohoku earthquake, where abnormally high accelerations > 1 g were recorded. Based on vertical array records, models of soil behavior are constructed at 28 sites in northern Honshu (Tohoku region). They are compared with previously studied models of soil behavior in southern Tohoku and Kanto regions, where shock waves were identified as possible causes of the recorded high accelerations. Shear moduli did not reduce during strong motion at many sites, and the behavior of softer and denser soils was similar to a large extent. The nonlinearity of soil response during the Tohoku earthquake was weaker than that observed earlier during the 1995 Kobe and 2000 Tottori earthquakes (Mw ~6.7–6.8). Instead, a widespread soil hardening was found, most expressed at stations recorded the highest PGAs. To explain the observed features of soil behavior, two possible mechanisms are suggested, such as, 1) shock wave fronts generated by rupture propagation along the fault plane induce soil hardening and high PGAs; 2) soil compaction and hardening is a soil response to long-lasting dynamic loadings during the earthquake. Most likely we may expect similar effects of soil hardening and generation of high PGAs during other mega-thrust earthquakes in future.

## Introduction

Records of the 2011 Tohoku earthquake made by strong motion networks K-NET and KiK-net remain the most interesting and informative for seismologists, because they describe strong motion in the near-fault zones during the largest mega-thrust earthquake to date. Nineteen stations recorded anomalous strong ground motions that we have never seen previously^1^, with peak ground accelerations (PGAs) exceeding 1 g and the maximum reaching ~3 g. Strong motion records made by seismic vertical arrays KiK-net are of great value because they allow us to study *in situ* soil behavior in detail, to better understand soil response and physical mechanisms of generation of abnormally high accelerations and finally reduce damage from strong earthquakes.

**Figure 1:**
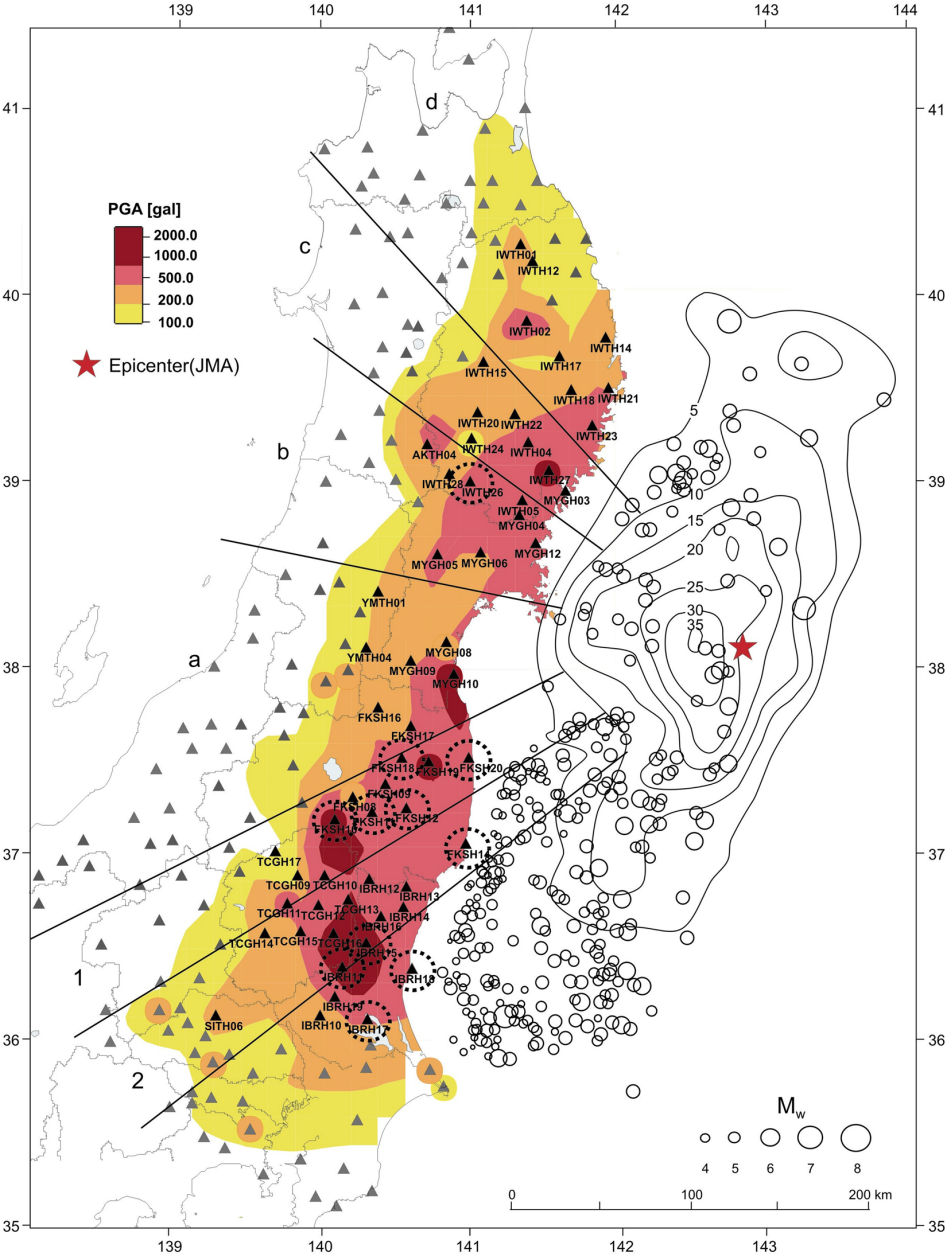
Map showing the distribution of PGAs recorded during the 2011 Tohoku earthquake^14^, locations of the main shock (star) and aftershocks (circles) (M > 3) recorded in 24 hours after the main shock, and KiK-net stations studied in this research – black triangles; other KiK-net stations – grey triangles. Contour lines depict slip distribution in meters, according to ‘joint source model’ by Koketsu *et al*.^23^. Dotted circles mark stations with the strongest manifestations of nonlinearity of soil behavior during the Tohoku earthquake. Numbers 1 and 2 and letters (**a**–**d**) indicate sectors discussed in the text (the figure was drawn by CorelDraw v. 11 based on the map provided by the website www.k-net.bosai.go.jp).

The network of seismic vertical arrays KiK-net was deployed in Japan after the catastrophic 1995 Kobe earthquake. Since 1996, the KiK-net strong-motion database grows and accumulates records of earthquakes of various magnitudes, including the 2011 mega-thrust Tohoku earthquake (Mw ~9.0). The distribution of PGAs in the near-fault zones reflects complicated patterns of seismic wave radiation and propagation from the extended source of the Tohoku earthquake (Fig. 1).

Based on the records of KiK-net vertical arrays, in^2^, models of soil behavior during the Tohoku earthquake were constructed for southern Tohoku and Kanto regions (to the south of FKSH17, 37.66 N parallel, Fig. 1), where abnormally high accelerations were recorded, i.e., vertical distributions of stresses and strains induced in soil layers during the strong motion were estimated. The models show that during the Tohoku earthquake, we did not observe a widespread soil nonlinearity, similar to that observed earlier during the 1995 Kobe and 2000 Tottori earthquakes (Mw ~ 6.7–6.8)^3–5^. Instead, at many sites soils experienced hardening during the Tohoku earthquake. The strongest nonlinearity of soil response and reduction of shear moduli were observed at coastal stations closest to the fault. At remote sites, where abnormally high PGAs were recorded, shear moduli in soil layers increased at the beginning of strong motion and reached their maxima at the moments of the highest intensity of strong motion, indicating soil hardening and then decreased with decreasing the intensity of motion^2^.

The explanation of these observations and features of soil behavior was suggested in^6^. A shock wave could be the force that compressed and hardened the soils at stations recorded the highest PGAs; the shapes of the velocigrams and seismograms at these stations supported this assumption. For the verification, the shapes of acceleration time histories were studied at KiK-net sites located in narrow sectors going from the source (1 and 2 in Fig. 1). Changes in the shapes of the acceleration time histories with distance from the source were found, such as, decrease of the duration and increase of the intensity of strong motion, which indicate phenomena similar to seismic wave overlapping (directivity effects) and shock wave generation at sites recorded the highest PGAs (FKSH10, TCGH16, IBRH11, and others). At larger distances from the source, PGAs sharply fall (at TCGH17, TCGH09, TCGH14, IBRH10 sites, Fig. 1). Shock wave fronts could compress and harden the soils and finally, produce high accelerations during the 2011 Tohoku earthquake^6^.

Actually, the observed peculiarities of soil behavior in southern Tohoku and Kanto regions during the Tohoku earthquake are inconsistent with our representations about typical soil behavior in strong ground motion, based on laboratory experiments^7^ and observations of *in situ* soil behavior during past strong earthquakes (for example^3–5,8^). Soil softening, reduction of shear moduli and increase of damping (and therefore, decrease of PGAs) were considered as typical features of soft soil behavior during strong ground motion. The computer codes for calculation of soil response in strong ground motion developed since 1970-th account for these features of soil behavior^9,10^ etc.

Later, in 2000s, a method was suggested for studying *in situ* soil behavior in strong ground motion in detail by constructing models of soil behavior based on vertical array records^3^. Models were constructed of soil behavior in the near-fault zones of the 1995 Kobe (Mw ~ 6.8) and 2000 Tottori (Mw ~ 6.7) earthquakes, i.e., vertical distributions of stresses and strains induced in soil layers by the strong motion were estimated^3,5^. Then models of soil behavior in the near-fault zones of the 1999 Chi-Chi earthquake (Taiwan, Mw = 7.6) were constructed^11^, where the input motion to soil layers was simulated by finite-fault stochastic method. Based on these studies, the conclusions were drawn about ‘typical’ soil behavior in strong ground motion (Mw ~ 6.7–7.6):

(1) Near the fault plane (within ~1/4 *L*, where *L* is the length of the fault) the behavior of soft soils (*Vs* < 300 m/s) is essentially nonlinear and characterized by various types (soft or hard) of stress-strain relations, depending on the soil composition, depth, and saturation with water. Rheological properties of subsurface soft soils change during strong motion; with approaching the fault plane, the effect increases and involves deeper layers. The degree of nonlinearity of soil response decreases with distance from the fault, and at distances ~ *L* soil behavior becomes virtually linear. (2) Shear and compression moduli of soft soils reduce during strong motion; near the fault their reduction reaches ~ 80–90% of the initial values, and the recovery begins at the end of strong motion. (3) Nonlinearity of soil response attenuates high-frequency components of motion and strengthen low-frequency components, so that spectra of motion on the surface tend to take the form *E(f) ~ f*^*−n*^. (4) The behavior of non-cohesive soils at sites with the underground water level of ~10 m or less during strong motion is described by hard-type stress-strain relations; in such soils strong motion is usually amplified^12^.

The constructed models of soil behavior during the Tohoku earthquake showed ‘atypical’ soil behavior different from soil behavior observed during previously studied earthquakes^2^. Really, abnormally high PGAs recorded during the Tohoku earthquake are poorly compatible with substantial nonlinearity of soil behavior associated with energy losses.

In this paper, we continue studying soil behavior during the Tohoku earthquake that was started in^2,6^. Soil behavior is studied at KiK-net sites located in the near-fault zones in northern Honshu (Tohoku region). Models of soil behavior are constructed for 28 KiK-net sites, and changes of shear moduli in soil layers during strong motion are estimated. The degrees of nonlinearity of soil response during the Tohoku earthquake at KiK-net sites are also estimated. In northern Honshu high PGAs exceeding 1 g were recorded, and in this paper, the shapes of acceleration time histories in northern Honshu and their changes with distance from the fault are studied, to check for directivity effects, similar to those contributed to high PGAs in southern Tohoku and Kanto regions.

## Data and Method

Models of soil behavior are constructed for 28 KiK-net sites located in northern Honshu (to the north of FKSH17, 37.66 N parallel). The KiK-net sites are located at epicentral distances of 137–271 km, and the recorded PGAs reach ~135–1136.8 Gal on the surface and ~34–241 Gal at depth. Table 1 provides information on these stations, such as, the coordinates, soil conditions, epicentral distances, recorded PGAs on the surface and at depth, depths of the boreholes. The last columns contain the estimates of the degrees of nonlinearity of soil response, described below.

**Table 1:** Information on the KiK-net stations in northern Honshu, for which models of soil behavior during the Tohoku earthquake were constructed.

Site code	Latitude (°)	Longitude (°)	Subsurface soils: (d/V_s_)	V_s30_ (m/s)	Epic distance (km)	PGA on surf (Gal)	PGA at dep (Gal)	Borehole depth (m)	*nl*_1_, %	*nl*_2_, %	*nl*,%
MYGH12	**38.64**	**141.44**	**6/280**	**748**	**137**	**568.3**	**241**	**105.5**	**0**	**1**	**1**
MYGH03	38.92	141.64	4/350	934	140	511.4	154	120	0	2	1
MYGH04	38.79	141.33	4/220	850	154	632.0	122	103	0	4	2
IWTH27	39.03	141.53	4/150	670	155	863.6	108	103	0	4	2
IWTH05	38.87	141.35	2/160	429	156	791.3	159	103.3	1	1	1
IWTH23	39.27	141.82	4/370	923	158	521.0	149	106	0	3	2
MYGH06	38.59	141.07	2/200	593	165	327.8	168	103	0	13	7
IWTH21	39.47	141.93	2/150	521	172	464.3	75	103	0	8	4
MYGH10	**37.94**	**140.89**	**3/175**	**343**	**174**	**1136.8**	**219**	**208**	**0**	**22**	**11**
IWTH04	**39.18**	**141.39**	**5/220**	**456**	**175**	**530.0**	**87**	**109**	**9**	**17**	**13**
MYGH08	**38.11**	**140.84**	**1/20**	**303**	**177**	**301.1**	**209**	**103**	**0**	**10**	**5**
IWTH18	39.46	141.68	2/180	892	183	363.2	68	103	0	1	1
IWTH26	**38.97**	**141.00**	**4/130**	**370**	**188**	**615.2**	**116**	**110.5**	**13**	**23**	**18**
MYGH05	**38.58**	**140.78**	**2/120**	**305**	**189**	**503.1**	**179**	**340**	**9**	**7**	**8**
IWTH22	**39.33**	**141.30**	**10/262**	**532**	**192**	**346.0**	**73**	**103**	**24**	**8**	**16**
MYGH09	**38.01**	**140.60**	**2/150**	**358**	**198**	**362.3**	**127**	**103**	**0**	**6**	**3**
IWTH14	39.74	141.91	2/170	816	200	300.0	49	103	0	6	3
IWTH28	39.01	140.86	4/240	417	201	303.9	67	267	0	26	13
IWTH24	39.20	141.01	2/180	486	202	202.3	113	153.1	0	5	3
FKSH17	37.66	140.60	6/180	544	205	321.7	81	103	0	7	4
IWTH20	**39.34**	**141.05**	**2/110**	**289**	**209**	**407.9**	**192**	**159**	**0**	**18**	**9**
FKSH16	37.76	140.38	4/180	532	221	354.2	86	303	0	11	6
AKTH04	39.17	140.71	2/150	459	222	523.3	42	103	0	12	6
YMTH04	**38.08**	**140.30**	**6/130**	**248**	**225**	**263.4**	**49**	**103**	**0**	**5**	**3**
IWTH15	**39.61**	**141.09**	**4/150**	**338**	**227**	**220.6**	**69**	**125**	**0**	**13**	**7**
IWTH02	**39.83**	**141.38**	**5/150**	**390**	**230**	**744.2**	**34**	**105.5**	**0**	**12**	**6**
IWTH12	**40.15**	**141.42**	**8/230**	**368**	**259**	**291.7**	**49**	**103**	**21**	**5**	**13**
IWTH01	**40.24**	**141.34**	**10/222**	**438**	**271**	**353.0**	**39**	**203**	**0**	**9**	**5**

The method for constructing models of soil behavior during strong ground motion based on records of seismic vertical arrays is described in detail in many publications, such as^2–5^, and therefore, here we can give shorter description. We calculate the propagation of vertically incident shear waves (records of the deep device of a vertical array) in the overlying soil layers, and we compare the calculated and observed motions on the surface. We use the algorithm of nonlinear analysis by Joyner and Chen^10^ modified in such a way that, to describe the behavior of soil layers in strong ground motion, instead of one stress-strain relation we can use more relations to describe soil behavior more accurately and better satisfy observations on the surface. We account for differences in the behavior of different layers, depending on their composition, depth, and saturation with water, and we account for temporal changes in soil behavior (softening, liquefaction, etc.).

Based on the profiling data and the available information about soil behavior, we select reasonable stress-strain relations that best fit observations on the surface. As a result, we obtain simulated acceleration time histories approximating the observed ones and the corresponding models of soil behavior, i.e., vertical distributions of stresses and strains induced in soil layers by the strong motion. The models visualize soil behavior and allow us to estimate stresses, strains, shear moduli in soil layers, and the degree of nonlinearity of the soil response.

To construct models of soil behavior during the Tohoku earthquake, the profiling data at KiK-net sites provided by NIED were used, such as, the composition and thickness of soil layers, *P*- and *S*- wave velocities. The densities, shear stresses in failure *τ*_*max*_, and attenuation in the layers were estimated based on the empirical relationships and our past experience. Series of parametric nonlinear stress-strain curves were generated and tested. The best-fit stress-strain curves for the soil layers were selected by estimating the deviations of the simulated records from the observed ones. The deviations included ‘point-by-point’ differences of the records, differences in PGAs, intensities, and spectral compositions.

A rather good agreement between the observations and simulations at the studied KiK-net sites was obtained, when the behavior of all soil layers during the Tohoku earthquake was described by one normalized stress-strain curve of a hard type. To account for temporal changes in soil behavior, the input motion was divided into 5-second time intervals, and calculations were performed successively, interval by interval, altogether, 32 intervals, or 160 s of the strong motion.

The constructed models of soil behavior were used to estimate variations of shear moduli in soil layers during the Tohoku earthquake. The models of soil behavior represent groups of hysteretic stress-strain curves that describe cycles of loading and unloading of soils during strong motion in 5-second time intervals. Shear moduli were calculated as the ratios of mean stresses (in relative units) to mean strains (in relative units) estimated based on the hysteretic curves in 5-second intervals. The obtained estimates of shear moduli (in relative units) were then averaged over all soil layers at each of the studied KiK-net stations.

Finally, to conclude about the nonlinearity of soil response during the Tohoku earthquake, the degree of nonlinearity of soil response at the studied sites was quantitatively estimated. We can estimate the degree of nonlinearity of soil response by comparing borehole transfer functions calculated for weak motions *G*_*W*_*(f)*, when soil response can be considered as linear, and for the Tohoku earthquake *G*_*T*_*(f)*. The approach is similar to that proposed by Field *et al*.^13^, who compared linear and nonlinear amplification functions applied to the 1994 Northridge earthquake. The transfer functions were calculated for series of weak earthquakes preceding the Tohoku earthquake. For each station, 20 records of weak earthquakes occurred in the 2000s were selected, for which PGAs on the surface did not exceed 5–10 Gal. As known^13^, in such conditions the behavior of soils is virtually linear. For these weak events, the average transfer functions and their confidence limits were calculated for the studied 28 KiK-net sites.

The method for estimation of the extent of nonlinearity of soil response is based on our representations of nonlinear wave effects in soil layers. As known, the propagation of seismic waves in soil layers is accompanied by nonlinear wave effects, such as, interaction of spectral components of the waves, nonlinear damping, higher harmonic generation, etc. The energy of the propagating waves is redistributed over the spectra so that spectral components at high and medium frequencies weaken, low-frequency components slightly amplify, and spectra of motion on the surface tend to take the form *E(f) ~ f* ^*−n*^^12^. Thus, nonlinearity influences both the amplification and frequency content of seismic waves propagating in soil layers. The difference in these parameters calculated for weak and strong motions can be considered as the estimate of the degree of nonlinearity of soil response during strong motion (another way to estimate the degree of nonlinearity of soil response is provided by nonlinear identification method^4^). We can obtain this estimate by comparing borehole transfer functions calculated for weak and strong motions.

Nonlinearity of soil response during strong motion leads to a decrease in amplification of seismic waves propagating in soil layers, and this decrease *nl*_1_ can be estimated as the difference between the definite integrals of the transfer functions calculated for weak *G*_*W*_*(f)* and strong motions *G*_*T*_*(f)* in seismic frequency range normalized by weak-motion transfer functions *G*_*W*_*(f)*:

1 $$n{l}_{1}=\frac{{\int }_{{f}_{1}}^{{f}_{2}}({G}_{W}(f)-{G}_{T}(f))df}{{\int }_{{f}_{1}}^{{f}_{2}}{G}_{W}(f)df},$$

where *f*_1_ and *f*_2_ define the seismic frequency range within which the nonlinearity *nl* is estimated.

Nonlinearity of soil response during strong motion leads to changes in the frequency content of seismic waves propagating in soil layers, and these changes *nl*_2_ can be estimated by calculating the correlation coefficient between the transfer functions calculated for weak *G*_*W*_*(f)* and strong motions *G*_*T*_*(f)*. In this study, combined estimates of the degrees of nonlinearity of soil response were obtained, accounting for changes in the amplification and in the frequency content of seismic waves propagating in soil layers.

## Results and Discussion

The best-fit stress-strain relations in soil layers were found, and models of soil behavior were constructed at 32 5-second time intervals (160 seconds of strong motion) for 28 KiK-net sites located in northern Honshu (Tohoku region). At these sites, acceleration time histories contain two groups of intense waves associated with at least two large ruptures on the fault plane. At stations located in southern parts of Tohoku region, the second group of waves is more intense, whereas at stations located in northern parts of Tohoku region the intensities of the two groups of waves are similar.

**Figure 2:**
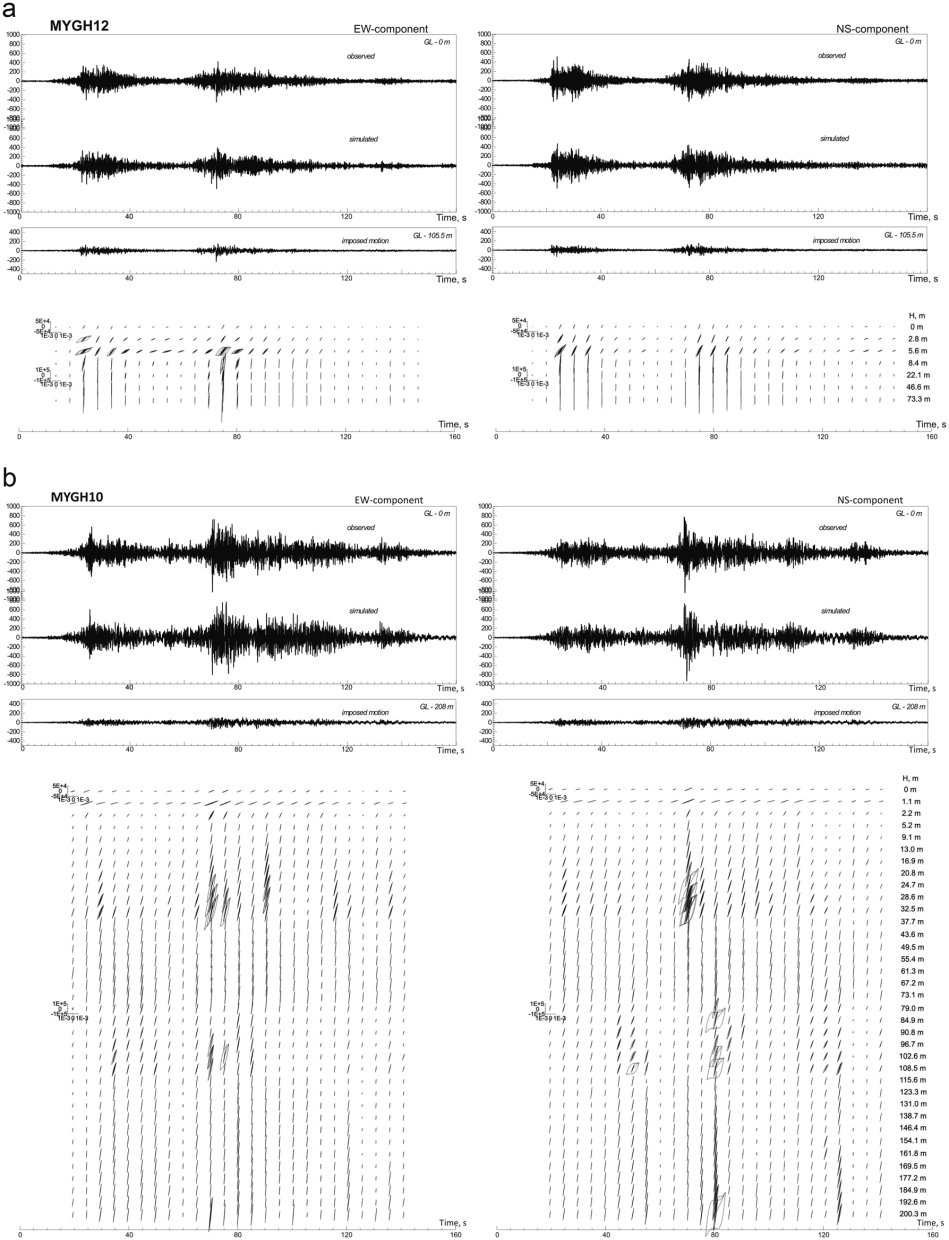
The acceleration time histories of the Tohoku earthquake, observed and simulated, and estimated stress-strain relations in soil layers, changing with time during strong motion: (**a**) at MYGH12 station; (**b**) at MYGH10 station. Accelerations are given in cm/s^2^, stresses are given in Pa, and strains in strains.

Examples of the constructed models showing the features of soil behavior in the studied area are presented in Fig. 2a,b and in Supplements, Fig. S1a–f. As seen from the figures, a rather good agreement was obtained between the calculations and the observations.

Soil conditions at the studied sites are described in Table 1 and in Supplements, Fig. S2a,b. Table 1 shows average *S*-wave velocities in the upper 30 meters, *V*_*S30*_ and the thicknesses *d* of the upper softer layers (*V*_*S*_ ≤ 300 m/s) with *S*-wave velocities in these layers *V*_*S*_. If a soil profile contains soft soils in the upper 30 m (*V*_*S30*_ < 370 m/s), or soft surface layers (*V*_*S*_ < 300 m/s) of a thickness *d* ~ 5 m or more, then at such sites we expect nonlinear soil behavior during strong motion. Such sites are considered as soft soil sites, and they are marked bold in Table 1. We found useful to identify such sites and discuss the features of soil behavior during the Tohoku earthquake at sites with softer and denser soils, as was done for KiK-net sites in southern Tohoku and Kanto regions^2^.

As seen from Figs. 2a,b and S1a–f, the constructed models indicate virtually linear soil behavior (the stress-strain relations are close to linear ones); significant nonlinearity is not observed. Figure S2a,b shows the estimates of maximum stresses and strains induced in soil layers by the strong motion and the profiling data at sites with softer (Fig. S2a) and denser soils (Fig. S2b). The stresses increase with depth, and the strains are maximal mostly in the upper layers (MYGH12, MYGH08, MYGH05, IWTH02, IWTH27, MYGH06, FKSH17, etc.).

Shear moduli were estimated at two horizontal components in 5-second time intervals; they are shown in Supplements, Fig. S3a,b for sites with softer and denser soils. We do not see ‘typical’ soft soil response during the Tohoku earthquake that was observed during past earthquakes (Mw ~ 6.7–7.6), such as, soil softening, reduction and recovery of shear moduli, though some sites show local drops of shear moduli (IWTH04, IWTH22, MYGH06, MYGH09, and IWTH24).

The obtained data show us a variety of models of soil response during the Tohoku earthquake at the studied sites; we will try to identify groups of stations with similar soil behavior.

At sites MYGH12 and IWTH05 located closely to the fault (~137–156 km) and recorded high PGA ~568–791 Gal (Table 1), shear moduli correlate with the intensity of motion; they grow and reduce twice, following two groups of intense waves (Fig. S3a,b). At other sites (MYGH04, IWTH21) the correlation is negative (Fig. S3b).

At sites MYGH10, IWTH20, YMTH04, shear moduli grow at the beginning and fall at the end of strong motion (Fig. S3a), whereas, at sites IWTH22 and IWTH04 we see the opposite: shear moduli reduce at the beginning and recover at the end of strong motion (Fig. S3a). Sites IWTH26, MYGH09 and IWTH24 show a slight decrease of the shear moduli during strong motion.

In the whole, the shear moduli remain rather high during the Tohoku earthquake (~0.8–1.0) at softer and denser soil sites (Fig. S3a,b), except MYGH08, MYGH09, and MYGH06 sites, where thick layers of very soft clays are deposited^14^. At many stations, variations of shear moduli indicate soil softening at some time intervals and soil hardening at others; generally, the behavior of softer and denser soils looks similar (Fig. S3a,b). Soil softening in strong ground motion is well known from laboratory and field experiments and from *in situ* observations: soft soils usually soften and loosen under strong motion, i.e., seismic velocities and shear moduli fall in soft soil layers, and absorption increases. Soil hardening is an opposite process, i.e. solidification and compaction of the soil, when seismic velocities and shear moduli grow during strong motion. The constructed models of soil behavior at the studied KiK-net sites and the estimates of shear moduli show us a widespread soil hardening during the Tohoku earthquake, such as, shear moduli at many soft soil sites grow or remain stably high (Fig. S2a,b), and soil behavior is virtually linear, with low absorption (Figs. 2a,b, S1a–f).

The last columns in Table 1 show the estimates of the degree of nonlinearity of soil response in percents. For comparison, similar estimates were obtained for KiK-net sites located in southern Tohoku and Kanto regions; they are presented in Table 2.

**Table 2:** Information on the KiK-net stations in southern Tohoku and Kanto regions.

Site code	Latitude (°)	Longitude (°)	Subsurface soils: (d/V_s_)	V_s30_ (m/s)	Epic distance (km)	PGA on surf (Gal)	PGA at dep (Gal)	Borehole depth (m)	*nl*_1_, %	*nl*_2_, %	*nl*,%
FKSH20	**37.49**	**140.99**	**28/350**	**357**	**178**	**663.0**	**356**	**112**	**35**	**8**	**22**
FKSH19	**37.47**	**140.72**	**20/255**	**338**	**201**	**914.0**	**350**	**103**	**18**	**14**	**16**
FKSH14	**37.03**	**140.97**	**8/166**	**237**	**205**	**481.4**	**123**	**150**	**40**	**26**	**33**
IBRH18	36.36	140.62	3/180	494	216	633.6	156	507	18	19	19
FKSH12	37.22	140.57	4/250	449	225	506.6	94	108	28	17	23
FKSH09	**37.35**	**140.43**	**10/244**	**585**	**230**	**562.6**	**188**	**203**	**0**	**13**	**7**
FKSH11	**37.20**	**140.34**	**34/241**	**240**	**244**	**504.7**	**186**	**118.2**	**13**	**34**	**24**
IBRH13	**36.80**	**140.57**	**16/269**	**335**	**249**	**570.8**	**128**	**103**	**21**	**10**	**16**
FKSH08	37.28	140.21	4/200	532	250	392.7	121	108	0	4	2
IBRH14	36.69	140.55	2/180	829	258	454.2	103	103	0	1	1
IBRH12	**36.84**	**140.32**	**6/240**	**486**	**265**	**782.4**	**121**	**203**	**8**	**20**	**14**
FKSH10	37.16	140.09	4/150	470	266	1335.4	180	203	13	21	17
IBRH16	**36.64**	**140.40**	**5/206**	**626**	**272**	**666.8**	**128**	**303**	**8**	**14**	**11**
FKSH18	**37.49**	**140.54**	**12/185**	**307**	**277**	**633.2**	**101**	**103**	**12**	**25**	**19**
TCGH13	36.73	140.18	4.5/150	546	282	907.5	142	143	9	22	16
IBRH15	36.5	140.3	2/60	450	284	1062.2	144	110	8	26	17
TCGH10	36.86	140.02	4/120	371	286	673.7	205	135	9	20	15
TCGH12	36.70	139.98	4/130	344	299	509.4	187	123	9	20	15
TCGH16	**36.55**	**140.08**	**18/163**	**213**	**301**	**1304.8**	**177**	**115**	**1**	**17**	**9**
TCGH09	**36.84**	**139.84**	**6/180**	**468**	**301**	**164.6**	**69**	**106**	**0**	**2**	**1**
IBRH11	**36.37**	**140.14**	**20/197**	**242**	**309**	**1223.9**	**267**	**106**	**31**	**23**	**27**
TCGH11	**36.71**	**139.77**	**12/198**	**329**	**314**	**469.6**	**61**	**203**	**0**	**6**	**3**
TCGH15	36.56	139.86	3/100	423	316	358.1	75	303	0	4	2
IBRH17	**36.09**	**140.31**	**10/212**	**301**	**318**	**522.8**	**118**	**514**	**30**	**10**	**20**
TCGH14	36.55	139.62	2/200	974	335	264.3	39	103	19	8	14
IBRH10	**36.11**	**139.99**	**20/110**	**144**	**338**	**334.4**	**98**	**903.5**	**19**	**5**	**12**
SITH06	**36.11**	**139.29**	**10/183**	**369**	**387**	**275.6**	**66**	**203**	**0**	**7**	**4**

**Figure 3:**
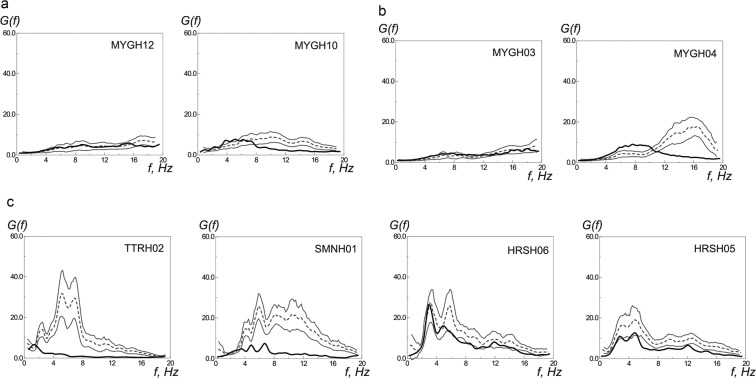
The borehole transfer functions at KiK-net sites calculated for the Tohoku earthquake *G*_*T*_*(f)* (black thick lines) and for weak motions *G*_*W*_*(f)* (thin dash lines – averages, thin solid lines – confidence limits): (**a**) at sites with softer soils MYGH12 and MYGH10; (**b**) at sites with denser soils MYGH03 and MYGH04; (**c**) the same at sites in the near-fault zones of the 2000 Tottori earthquake.

Figure 3a,b and Supplements, Fig. S4a–d show the borehole transfer functions at all the studied KiK-net sites calculated for weak motions and for the Tohoku earthquake. The transfer functions are estimated based on spectra calculated over the whole lengths of seismic motion during weak earthquakes and the Tohoku earthquake. As seen from preliminary calculations, the estimates of the degrees of nonlinearity based on the differences in amplification (in weak and strong motions) *nl*_1_ are more stable than the estimates based on the correlation of the transfer functions (in weak and strong motions) *nl*_2_, because they are less dependent on the smoothness of the transfer functions that affects the latter, *nl*_2_. Realistic estimates of the nonlinearity of soil response *nl* were obtained from the combination of *nl*_1_ and *nl*_2_, such as, *nl* = (*nl*_1_ + *nl*_2_)/2, where *nl*_1_ and *nl*_2_ were calculated based on the transfer functions taken at frequencies below *f* ~ 12 Hz. The *nl* estimates based mainly on low-frequency components (*f* < 12 Hz) are actually more sensitive to nonlinear effects, because spectra of seismic waves passed through soil layers tend to take the form *E(f)* ~ *f* ^*−n*^^12^, that is, the low-frequency parts of the spectra are more sensitive to nonlinear effects, because in spectra of seismic waves passed soil layers, the low-frequency components are amplified.

This technique for estimating *nl* was verified on records of the 2000 Tottori earthquake (Mw ~ 6.7), where near-fault sites TTRH02 and SMNH01 (located at 7 km and 8 km from the fault plane) showed strong nonlinearity of soil response^5,12^. At TTRH02 and SMNH01 sites, *nl* ~ 83% and *nl* ~ 69%, respectively. At remote sites HRSH06 and HRSH05 (57 km and 80 km from the fault plane), *nl* ~3% and ~9%. Figure 3c shows the transfer functions for these KiK-net sites recorded the 2000 Tottori earthquake.

The strong-motion transfer functions constructed for the 2000 Tottori and the 2011 Tohoku earthquakes clearly show the differences in the degrees of nonlinearity of soil behavior: high nonlinearity in the first case (Fig. 3c) and rather low nonlinearity in the second case (Figs. 3a,b, S4a–d). Figure 3a,b shows the transfer functions for stations located close to the source on soft (MYGH12 and MYGH10) and dense (MYGH03 and MYGH04) soils. As seen from the figure, during the Tohoku earthquake, the behavior of soft and dense soils is similar, i.e., soft soils do not show nonlinearity.

As seen from Table 1, the highest PGAs at depth (~179–241 Gal) were recorded at stations with softer soils MYGH12, MYGH10, MYGH08, MYGH05, and IWTH20. However, the high PGAs did not lead to high nonlinearity of the soil response at these sites; the degrees of nonlinearity are rather low, *nl* ~ 1–11%. The strongest nonlinearity is observed at soft soil sites IWTH26 (*nl* ~ 18%) and IWTH22 (*nl* ~ 16%). On the average, *nl* estimates are slightly higher on softer soils (~8.4%) than on denser soils (~3.9%).

In southern Tohoku and Kanto regions, PGAs at depth are generally higher than in northern Tohoku; they reach ~350–356 Gal, and the degrees of nonlinearity of soil response are also higher (Table 2), up to ~27–33%. The highest estimates (*nl* > 18%) are obtained at sites FKSH20, FKSH14, IBRH18, FKSH12, FKSH11, FKSH18, IBRH11, and IBRH17; in Fig. 1 these sites are marked by dash circular lines. The sites possessing higher nonlinearity of soil response are located on the coast, close to the source, and at rather large epicentral distances, and obviously, the nonlinearity of soil response is higher at sites with high PGAs at depth. Average *nl* estimates on softer soils are *nl* ~ 14.9%, which is slightly higher than that on denser soils, *nl* ~ 12.8%.

Note that in southern Tohoku and Kanto regions, *nl* estimates tend to decrease with distance from the source (the highest *nl* estimates were obtained at sites located close to the fault), whereas in northern Honshu, we do not see this tendency: *nl* estimates first grow with distance (at sites MYGH12 and MYGH10 we observe soil hardening and virtually linear soil behavior), and then decrease. This evidently indicates different mechanisms of soil response at the two groups of stations, more complicated and diverse in northern Honshu.

Finally, the waveforms of acceleration time histories in northern Honshu are studied, to check for directivity effects similar to those found in southern Tohoku and Kanto regions that apparently led to generation of high PGAs. In^6^ the waveforms of the acceleration time histories of the Tohoku earthquake in narrow sectors (1 and 2 in Fig. 1) were examined, and decrease of the duration and increase of the intensity of strong motion with distance from the source were found that indicate overlapping of seismic waves and formation of shock wave fronts. Last decades, similar phenomena were observed during some other strong earthquakes possessing large sources, where the cracks ruptured the fault planes at supershear speeds ^15–21^. In southern Tohoku and Kanto regions, a shock wave could be produced by the rupture propagating in the south-west direction on the fault plane of the Tohoku earthquake at a sub-Rayleigh speed^6^.

Records of the Tohoku earthquake made by deep accelerometers (to eliminate the soil response) of KiK-net vertical arrays located in sectors a, b, c, and d (Fig. 1) are shown in Supplements, Fig. S5a–d. In sectors a and d, the intensity of motion and PGA values decrease with distance from the source, and the shapes of the acceleration time histories are rather stable, whereas in sectors b and c, PGAs do not definitely decrease with distance, and we can notice some decrease of the duration of strong motion in the first group of waves in sector b. Thus, the directivity effects are likely in northern Honshu in the first group of intense waves (Fig. S5b,c).

The first group of intense waves is associated with one of the main ruptures on the fault plane of the Tohoku earthquake^22^. According to many authors, the source models of the 2011 Tohoku earthquake describe a large rupture area of about 200 × 500 km and a long-duration source process (~150 s)^22^. The isochrons shown in Fig. 1 reflect slip distribution on the fault plane according to the ‘joint’ source model by Koketsu *et al*.^23^ constructed through joint inversion of teleseismic, strong motion, geodetic, and tsunami datasets. The authors conclude that the earthquake consisted of three main ruptures corresponding to the zones of large slips on the fault plane. After a small rupture in the initial 50 s, the first rupture expanded at a speed of ~1.8 km/s to the northeast and east; 20 s later the second rupture began to propagate westward at a speed of ~1.5 km/s and became dominant with the final slip of 36 m. The third rupture then played the leading role, propagating southward at a speed of 2.5 km/s^3^.

The large second rupture on the fault plane propagated in western direction according to^23^ is responsible for the first group of intense waves recorded by northern stations. This rupture is clearly seen from tsunami waveform data as a ‘very large (~25 m) slip on the deep plate interface near Miyagi Prefecture’^24^, and the directivity effects (in sector b) can be associated with this rupture. These directivity effects could lead to soil hardening (during the passage of the first group of waves) in sector b (similar to what was observed in southern Tohoku and Kanto regions and described in^2^), at sites MYGH12, MYGH04, IWTH05, IWTH26, MYGH05, where shear moduli in soil layers reached 0.8–1.2 (Fig. S3a,b).

In general, the features of soil behavior during the Tohoku earthquake in northern Honshu, as well as in southern Tohoku and Kanto regions^2^, are widespread soil hardening (soil softening occasionally also took place), rather high shear moduli in softer and denser soils (Fig. S3a,b), and weak nonlinearity of soil response (Fig. S4a,b).

In northern Honshu, soil behavior is more diverse than in southern Tohoku and Kanto regions, apparently due to more complicated waveforms of acceleration time histories, i.e., two groups of intense waves instead of one group in southern Tohoku and Kanto regions. Obviously, one of the mechanisms of the observed soil hardening at sites in sector b (during the passage of the first group of waves) may be directivity effects, associated with a large rupture on the fault plane, as described above. However, high values of shear moduli at many sites in the near-fault zones (during the passages of the two groups of intense waves) suggest that there may be other mechanisms that lead to soil compaction and hardening.

The mega-thrust Tohoku earthquake caused long-duration ground motions, when soils experienced long-term dynamic loadings. Compaction and hardening of soils can be the soil response to these long-lasting dynamic loadings. As known, compaction is considered as a typical response of non-cohesive soils to dynamic loadings of moderate stress amplitudes^25^, which is due to fundamental physical laws associated with the tendency of loose media to reduce their volumes. In this work, stresses induced in soil layers by the strong motion are estimated; they range from units to hundreds of kPa (Figs. 2a,b, S1a–f, S2a,b). As known, loading stresses of units to tens of kPa correspond to soil compaction mode, whereas higher stresses reaching hundreds of kPa usually correspond to soil loosening mode^26^. Thus, estimates of stresses in soil layers during strong motion obtained in this paper indicate that vibrational compaction is possible in soils in the near-fault zones during the Tohoku earthquake. Soil hardening could occur during the passage of the second group of intense waves associated with the large rupture on the fault plane moving in southern direction^22^, at sites IWTH04, MYGH03, MYGH04, IWTH27, IWTH23, IWTH21, and IWTH18, where shear moduli grew till the end of strong motion, showing continuous soil hardening (Fig. S3a,b).

At the same time we should admit that, despite the success in studying *in situ* soil response in strong ground motion, numerous laboratory and field experiments, there still remain insufficiently studied issues concerning soil behavior under dynamic loadings. We cannot always correctly predict the behavior of certain soils in certain loading conditions; in particular, the behavior of dry loose sands, or dense water-saturated sands under long-term dynamic loadings, the behavior of dense water-saturated sands in drained conditions, etc.^25^. Probably, data on strong ground motion from future strong earthquakes will help us in solving these issues.

## Conclusions

KiK-net vertical array records revealed the features of soil behavior in the near-fault zones during the Tohoku earthquake. Along with soil softening, soil hardening was observed at many KiK-net sites, most expressed at sites recorded the highest PGAs. Compaction and hardening of soils led to increase of shear moduli and linearity of soil behavior.

The mechanisms of soil hardening during the Tohoku earthquake are not clearly understood. One of the possible mechanisms is associated with the directivity effects of seismic wave radiation and propagation from the extended source and formation of shock wave fronts that compress and harden the soils. On the other hand, soil hardening could occur as a soil response to long-duration ground motions during the Tohoku earthquake.

Obviously, soil hardening contributed to generation of abnormally high PGAs during the Tohoku earthquake. Consolidated soils behaved more linearly and effectively amplified strong ground motion. Thus, soil behavior during this mega-thrust earthquake Mw 9 differs from previously studied soil behavior during weaker events of Mw ~ 6.5–7.6, and apparently, we may expect similar effects of soil hardening and generation of high PGAs during future mega-thrust earthquakes.

## Data and Resources

Seismograms and soil stratigraphic setting used in this study are provided by the National Research Institute for Earth Science and Disaster Prevention (NIED) in Japan, and can be obtained from the Kyoshin and Kiban-Kyoshin Networks at www.k-net.bosai.go.jp.

## Supplementary information

Supplementary information.

Supplementary information 2.
