# Supplementary material for: Features of soil behavior in the near-fault zones during the 2011 Tohoku mega-thrust earthquake Mw 9
Source: Sci Rep. 2020 May 26;10:8717. doi: 10.1038/s41598-020-65629-2 (PMC7250923; doi:10.1038/s41598-020-65629-2)
Supplement: Supplementary file 1 — Supplementary information. [file 41598_2020_65629_MOESM1_ESM.pdf]

Supplementary Information file for the article:

Features of soil behavior in the near-fault zones during the 2011 Tohoku mega-thrust earthquake Mw 9

Olga V. Pavlenko

Schmidt Institute of Physics of the Earth, Russian Academy of Sciences, B. Gruzinskaya 10, Moscow 123242, Russia

e-mail: olga@ifz.ru, phone: +7 499 254 9025, mobile: +7 915 333 0260

This file is provided to contain all the necessary Figures that could not be included in the main text due to space constraints.

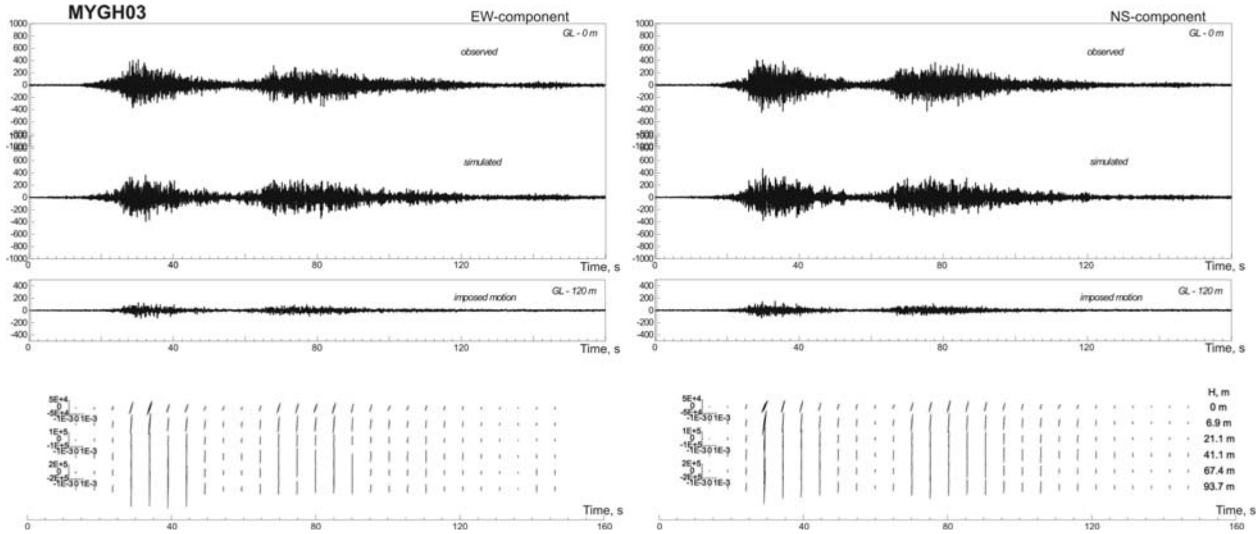

a

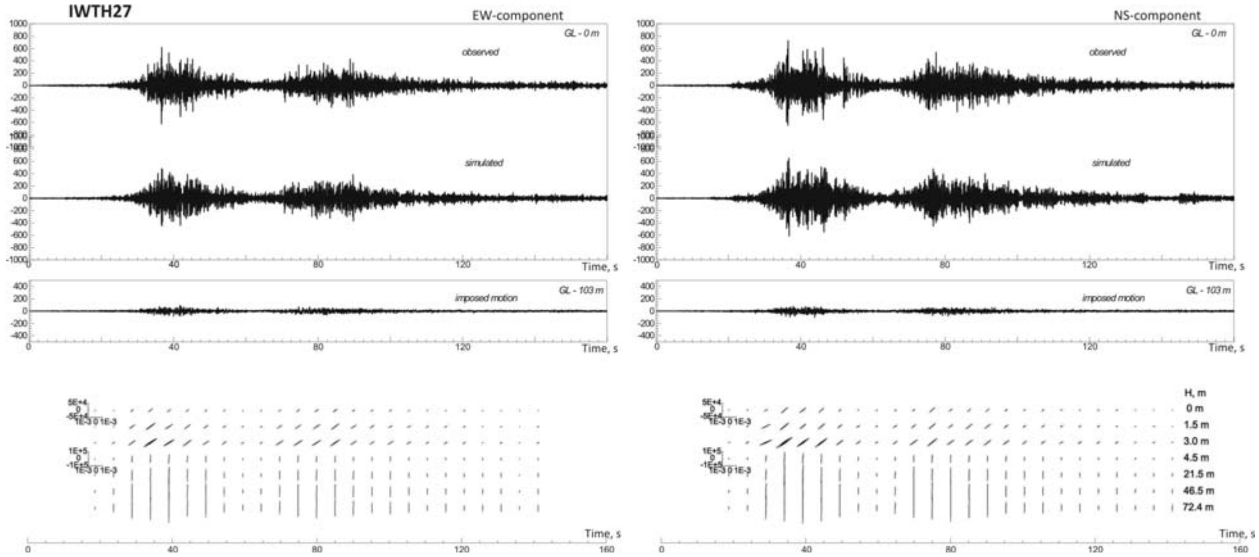

b

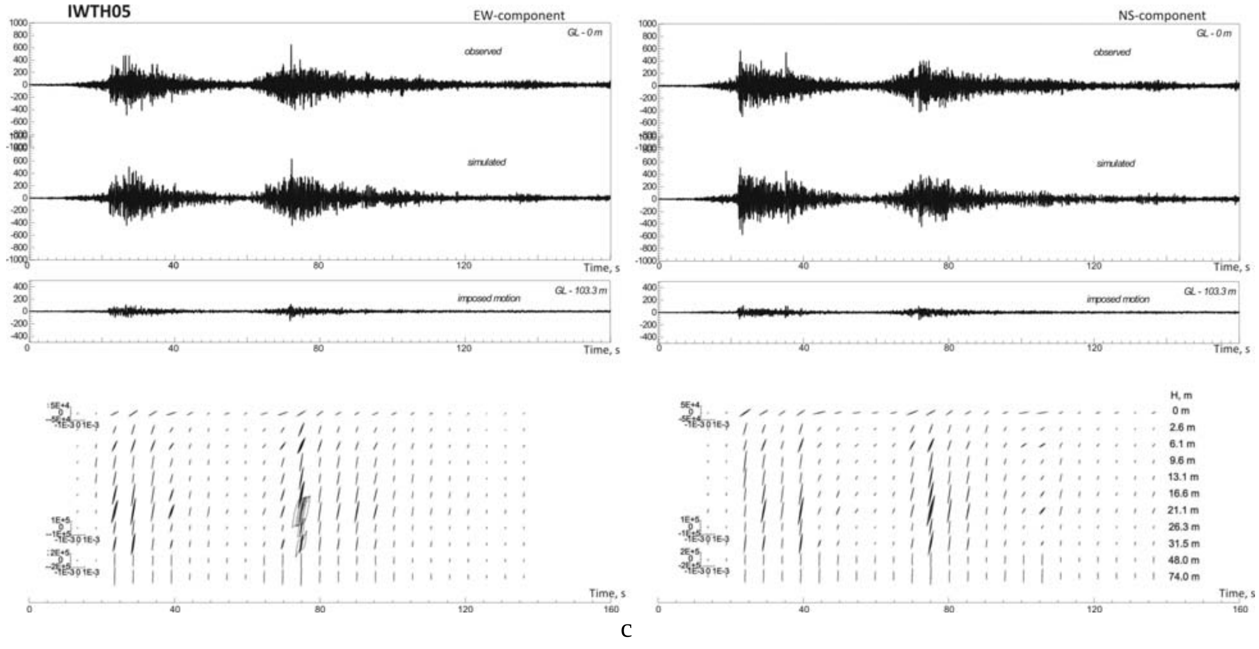

c

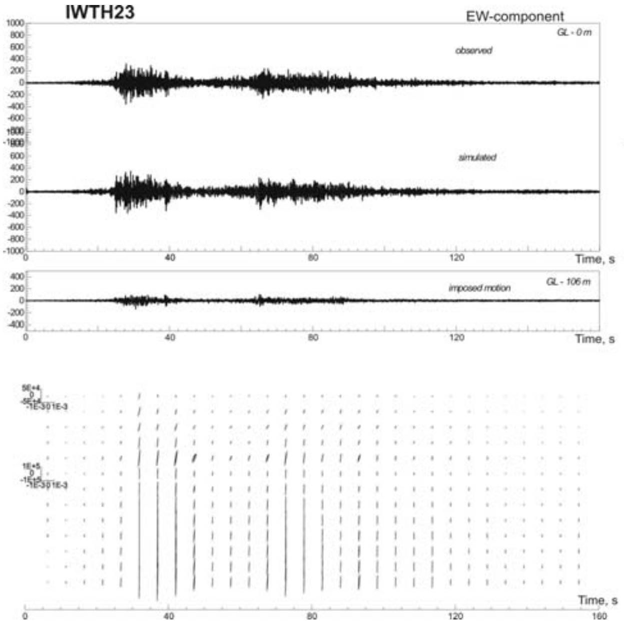
IWTH23
EW-component
NS-component
GL - 0 m
observed
simulated
imposed motion
GL - 106 m
Time, s
H, m
0 m
2.2 m
4.4 m
6.6 m
10.3 m
17.8 m
26.1 m
34.4 m
42.7 m
63.0 m
42.7 m
63.0 m
86.0 m

d

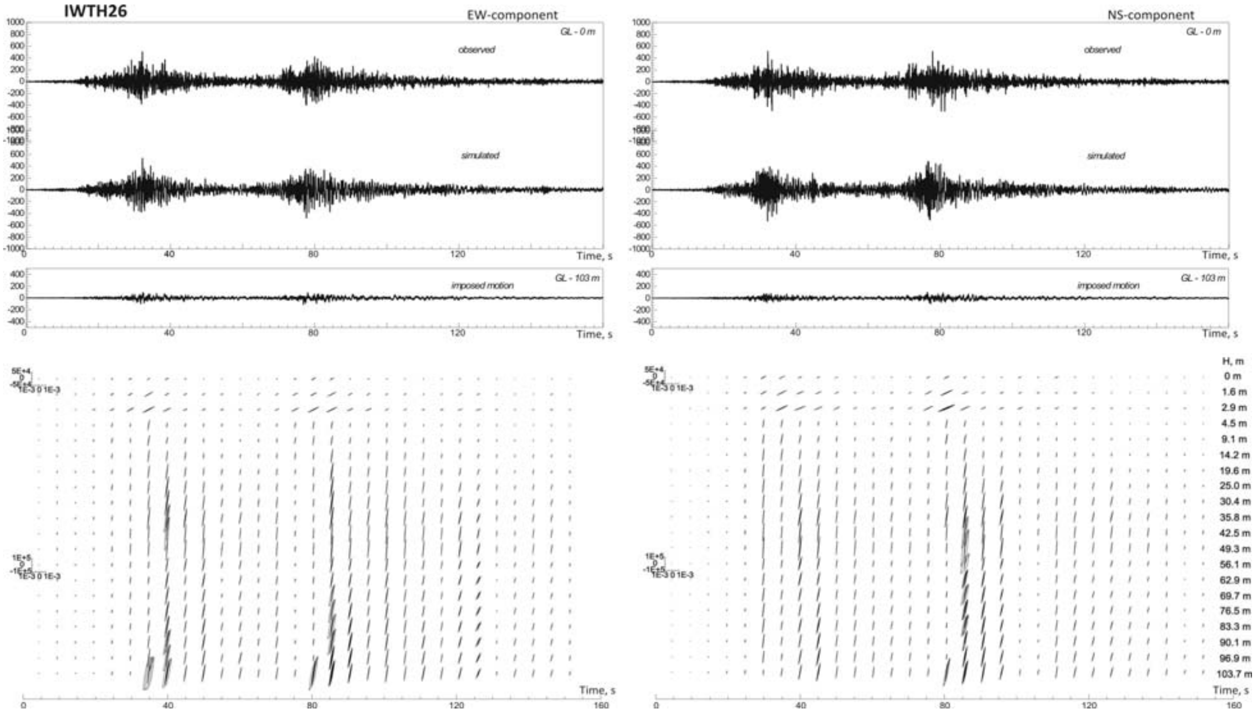
IWTH26
EW-component
NS-component
GL - 0 m
observed
simulated
imposed motion
GL - 103 m
Time, s
H, m
0 m
1.6 m
2.9 m
4.5 m
9.1 m
14.2 m
19.6 m
25.0 m
30.4 m
35.8 m
42.5 m
49.3 m
56.1 m
62.9 m
69.7 m
76.5 m
83.3 m
90.1 m
96.9 m
103.7 m

e

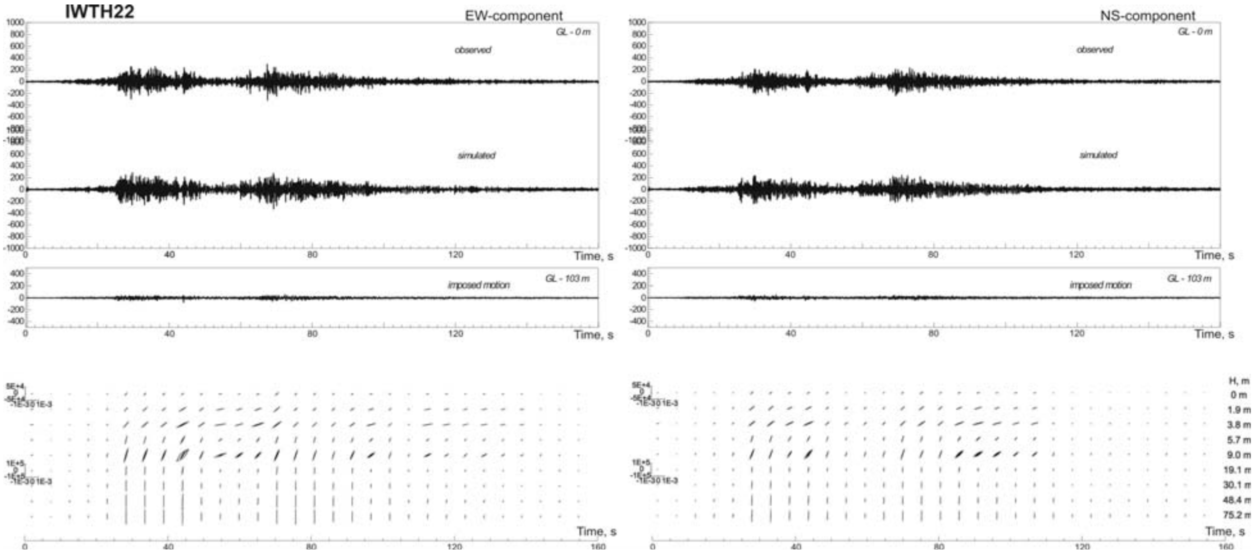
IWTH22
EW-component
NS-component
GL - 0 m
observed
simulated
imposed motion
GL - 103 m
Time, s
H, m
0 m
1.9 m
3.8 m
5.7 m
9.0 m
19.1 m
30.1 m
48.4 m
75.2 m

h

Figure S1. The acceleration time histories of the Tohoku earthquake, observed and simulated, and estimated stress-strain relations in soil layers, changing with time during strong motion: a - at MYGH03 station; b - at IWTH27 station; c - at IWTH05 station; d - at IWTH23 station; e - at IWTH26 station; f - at IWTH22 station. Accelerations are given in cm/s^2, stresses are given in Pa, and strains in strains.

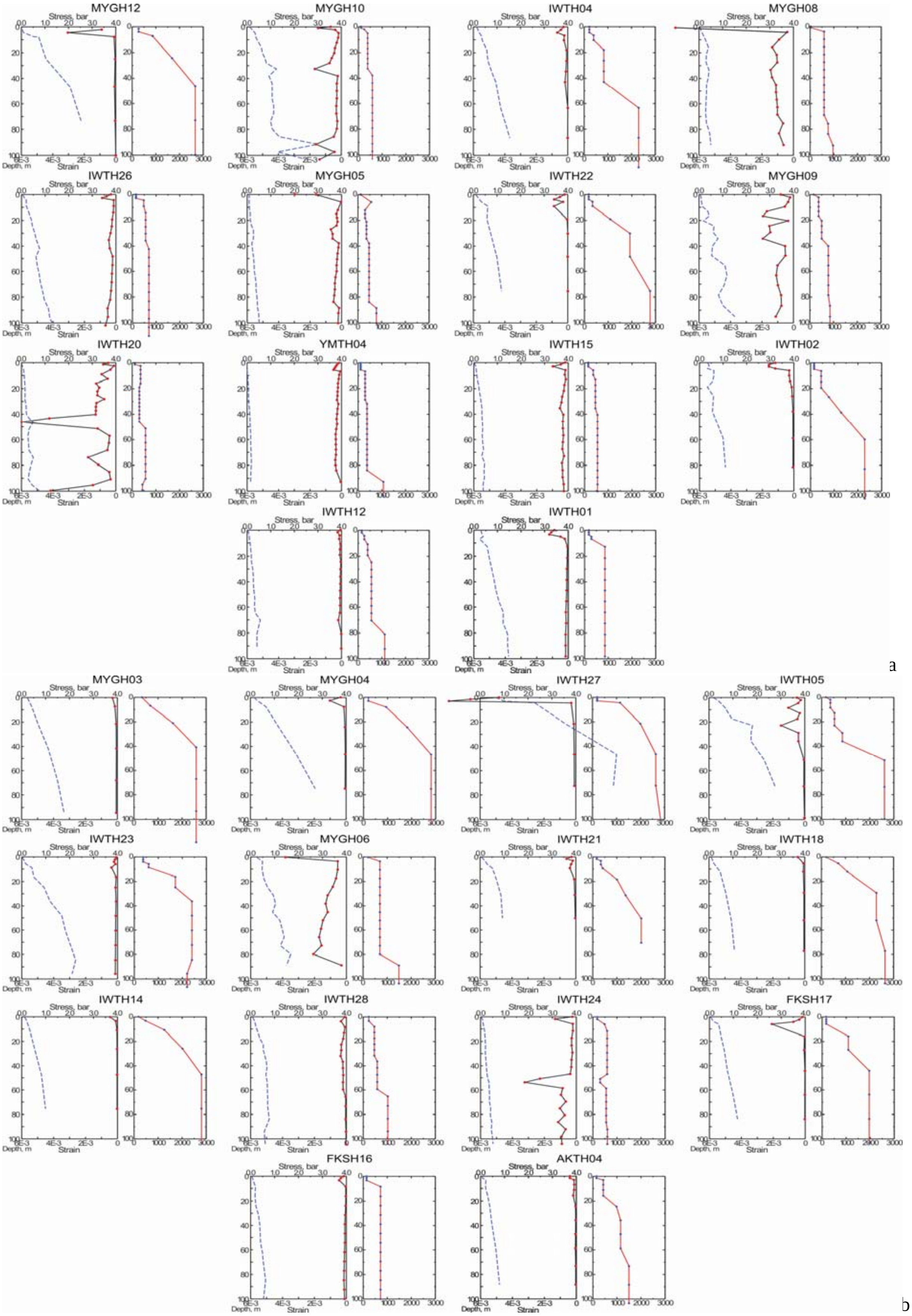

Figure S2. The profiling data: *Vs* velocities on the right, and the estimates of maximum stresses and strains (dash lines and solid lines, respectively, on the left) induced in the upper ~ 100 m by strong motion at the studied KiK-net sites: a – with softer subsurface soils, b – with denser subsurface soils. Stresses are given in bars, and strains in strains.

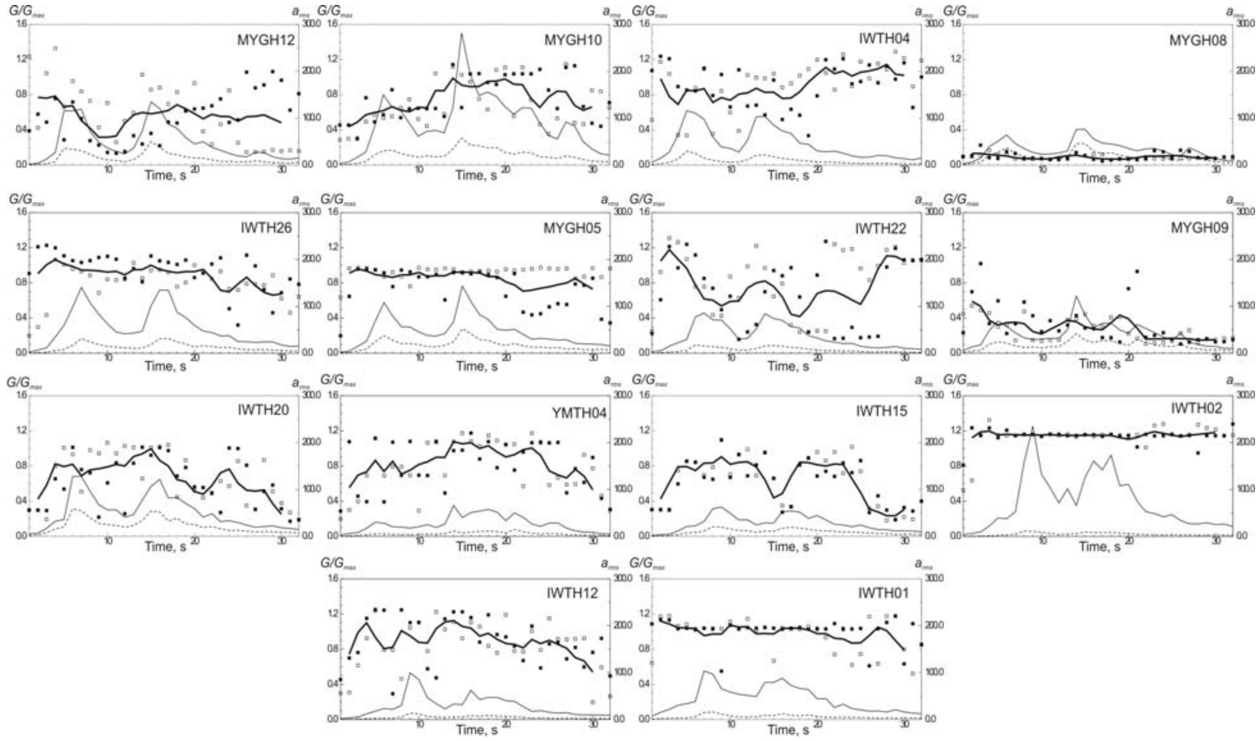

a

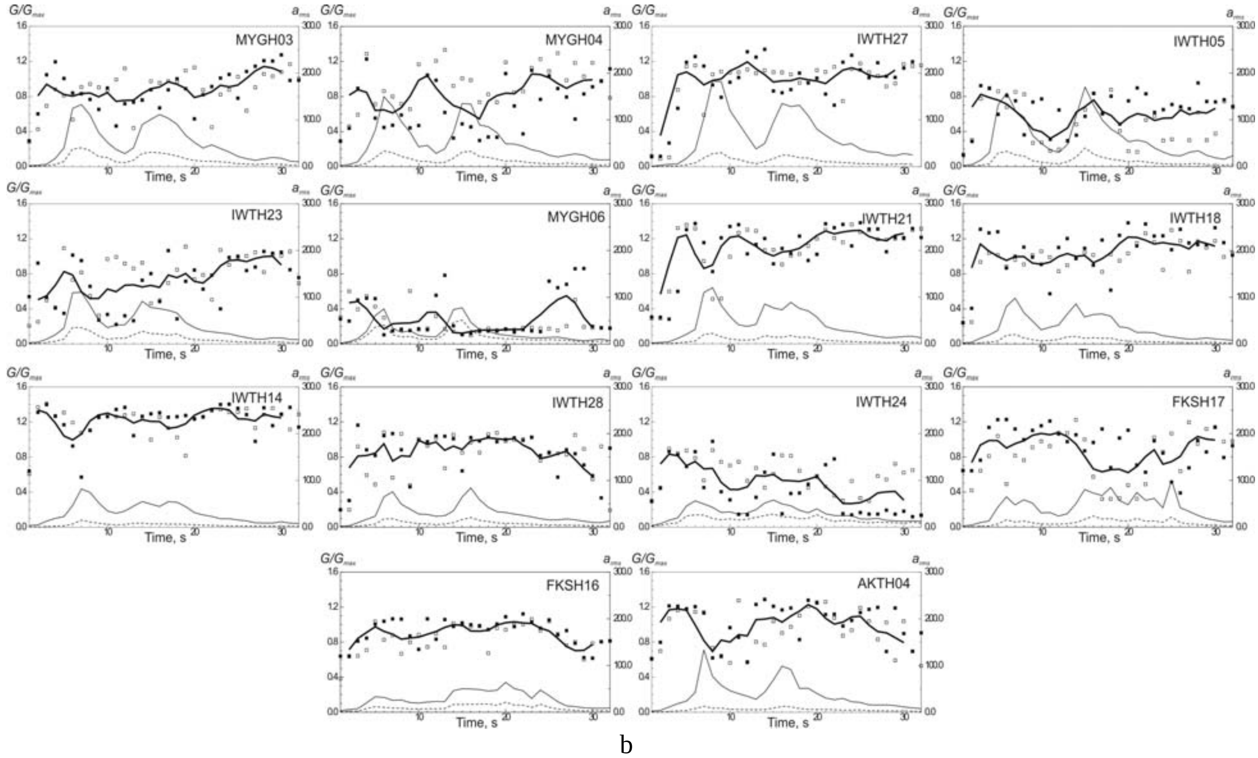

b

Figure S3. Changes of shear moduli in soil layers at the studied KiK-net sites during the Tohoku earthquake: a – at sites with softer subsurface soils, b – at sites with denser subsurface soils. Points indicate the estimated shear moduli at two horizontal components, thick lines show these values smoothed and averaged over two horizontal components. Thin lines are the intensities of motion on the surface (solid lines) and at a depth of location of the deep device (dash lines).

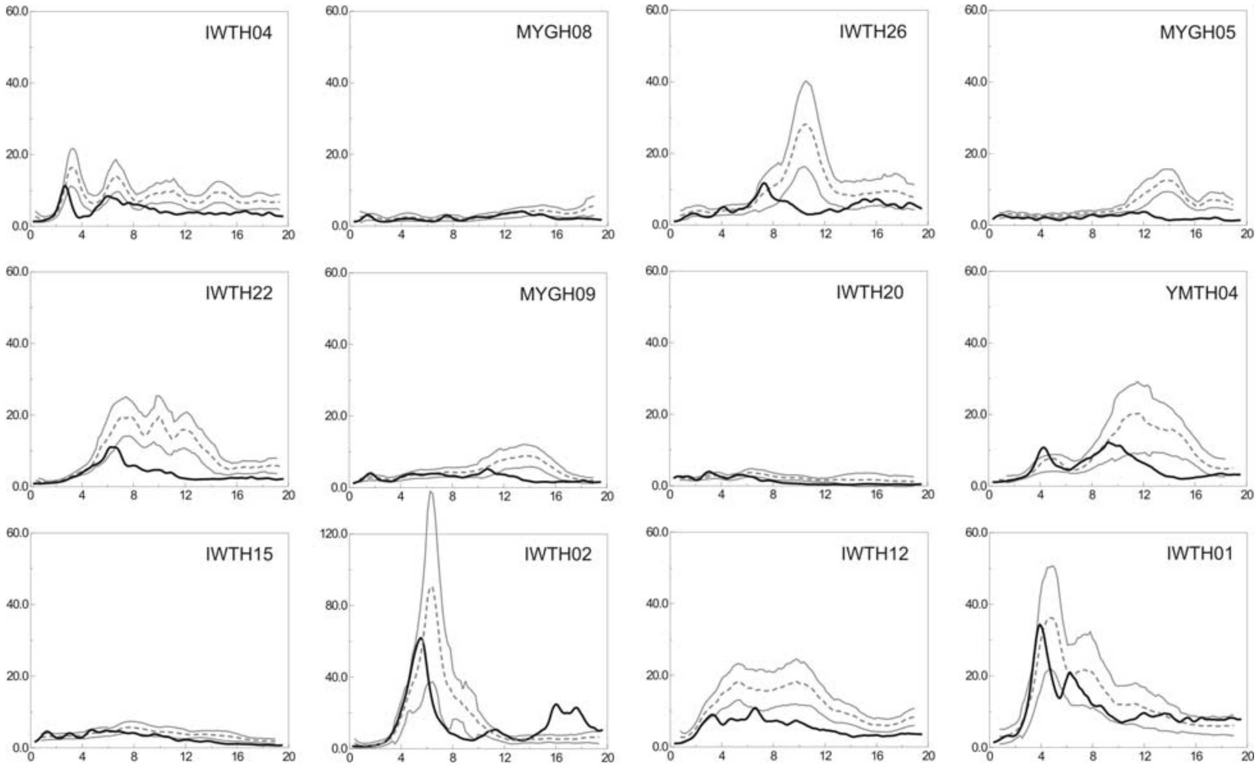
IWTH04
MYGH08
IWTH26
MYGH05
IWTH22
MYGH09
IWTH20
YMTH04
IWTH15
IWTH02
IWTH12
IWTH01

a

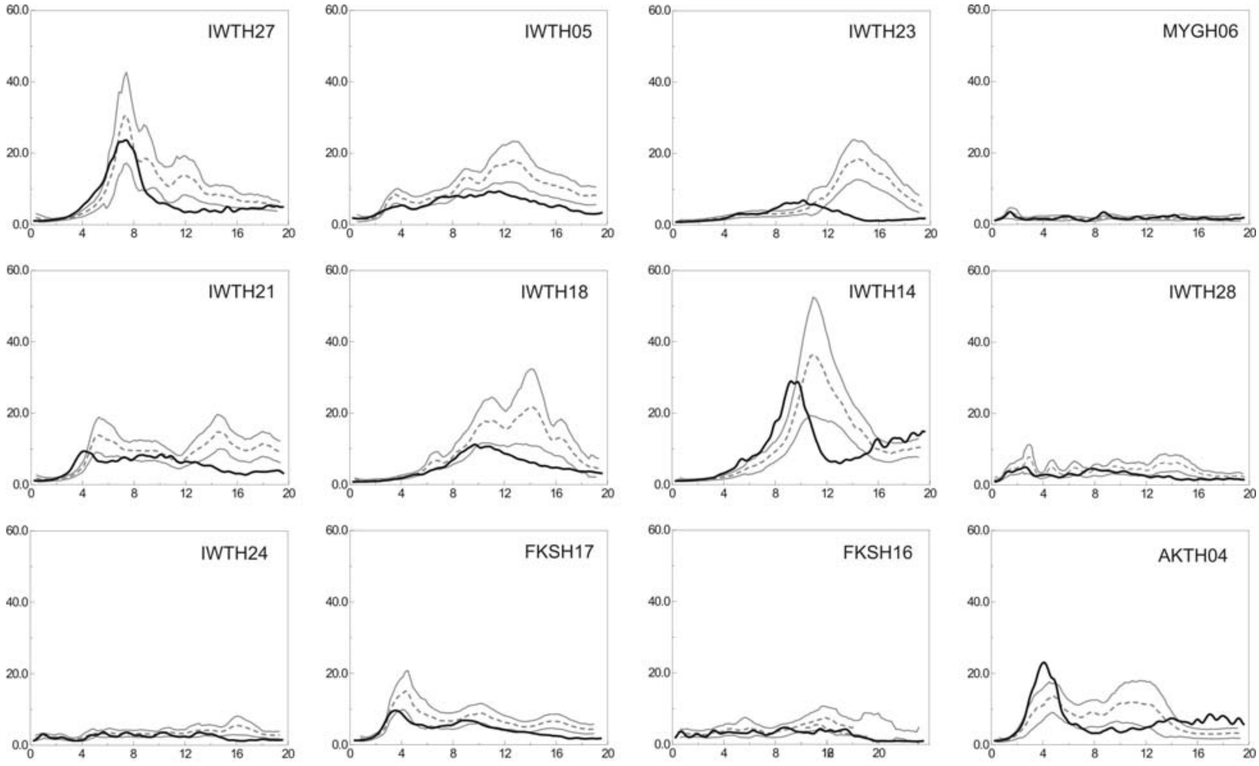
IWTH27
IWTH05
IWTH23
MYGH06
IWTH21
IWTH18
IWTH14
IWTH28
IWTH24
FKSH17
FKSH16
AKTH04

b

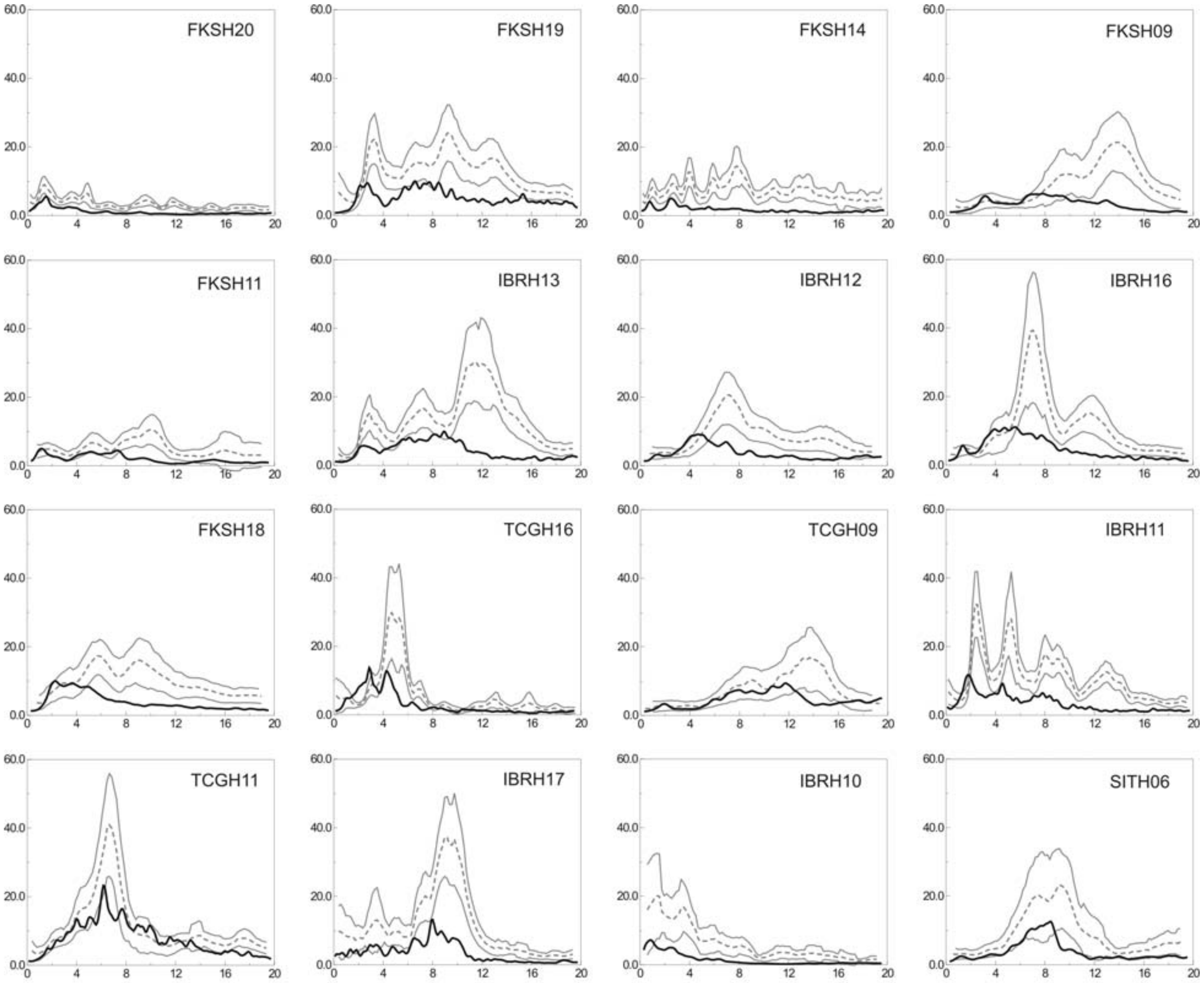

FKSH20
FKSH19
FKSH14
FKSH09
FKSH11
IBRH13
IBRH12
IBRH16
FKSH18
TCGH16
TCGH09
IBRH11
TCGH11
IBRH17
IBRH10
SITH06

c

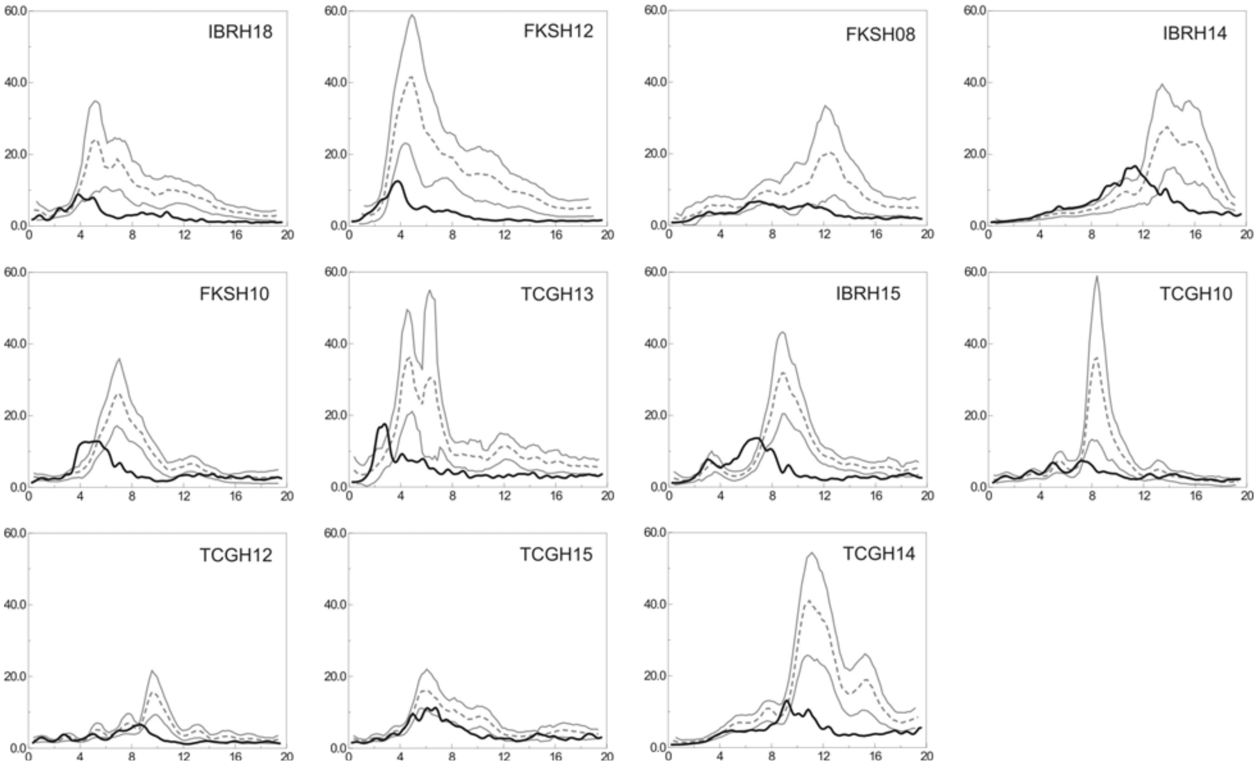

IBRH18
FKSH12
FKSH08
IBRH14
FKSH10
TCGH13
IBRH15
TCGH10
TCGH12
TCGH15
TCGH14

d

Figure S4. The borehole transfer functions at KiK-net sites calculated for the Tohoku earthquake $G_T(f)$ (black thick lines) and for weak motions $G_W(f)$ (thin dash lines – averages, thin solid lines – confidence limits): a – at sites with softer soils in northern Honshu; b – at sites with denser soils in northern Honshu; c – at sites with softer soils in southern Tohoku and Kanto regions; d – at sites with denser soils in southern Tohoku and Kanto regions. Abscissa is frequency.

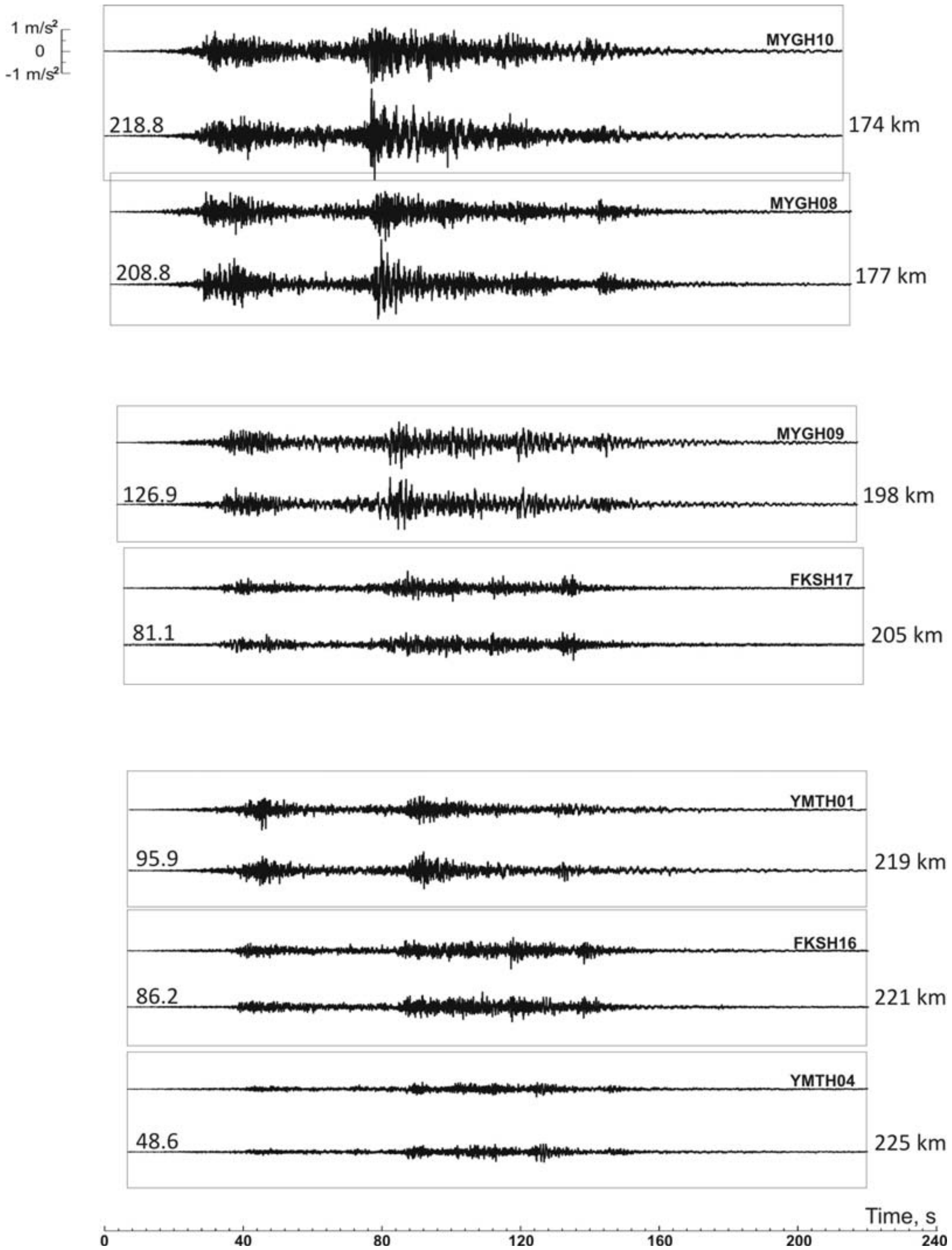

a

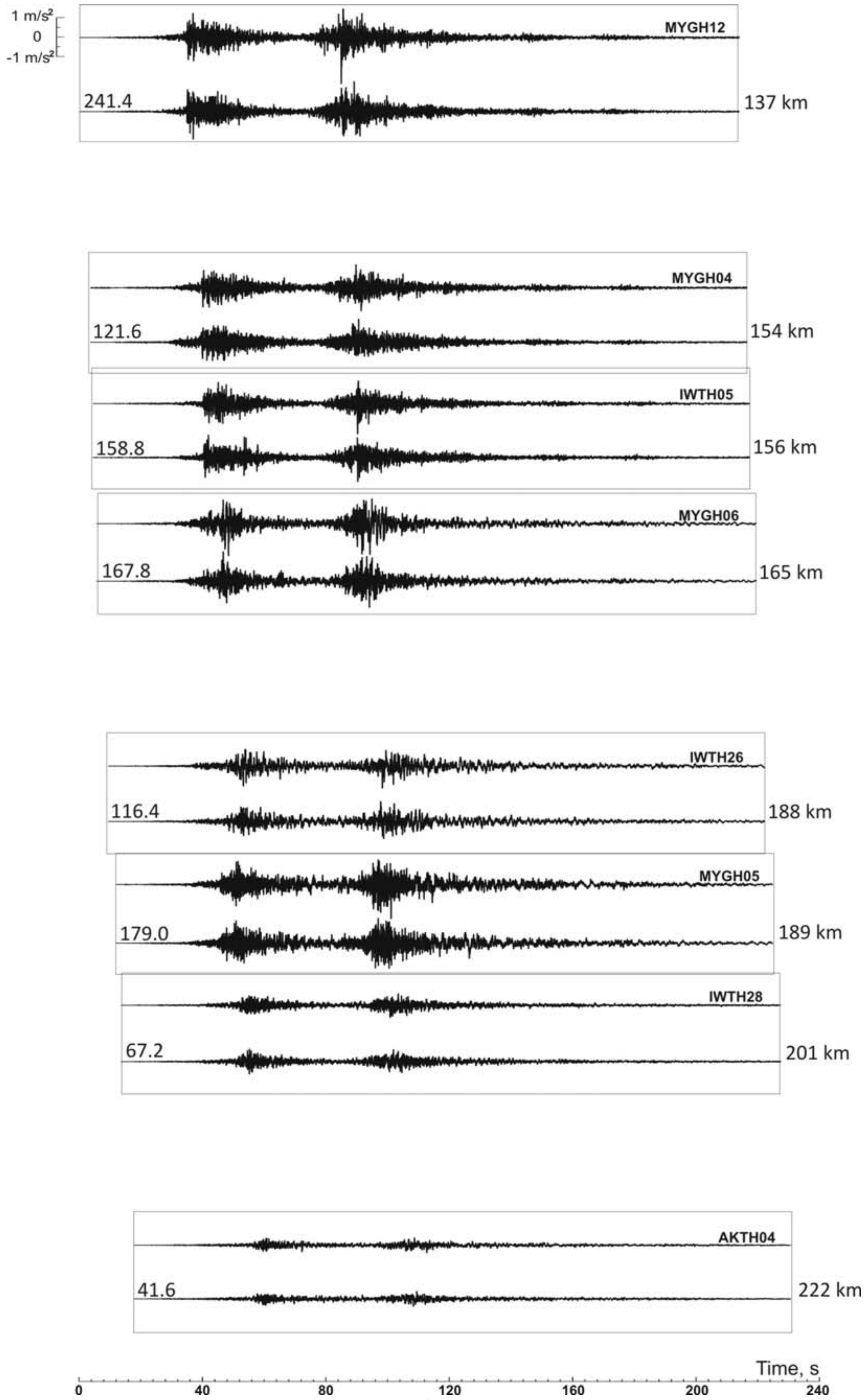
1 m/s²
0
-1 m/s²
MYGH12
241.4
137 km
MYGH04
121.6
154 km
IWTH05
158.8
156 km
MYGH06
167.8
165 km
IWTH26
116.4
188 km
MYGH05
179.0
189 km
IWTH28
67.2
201 km
AKTH04
41.6
222 km
Time, s
0
40
80
120
160
200
240

b

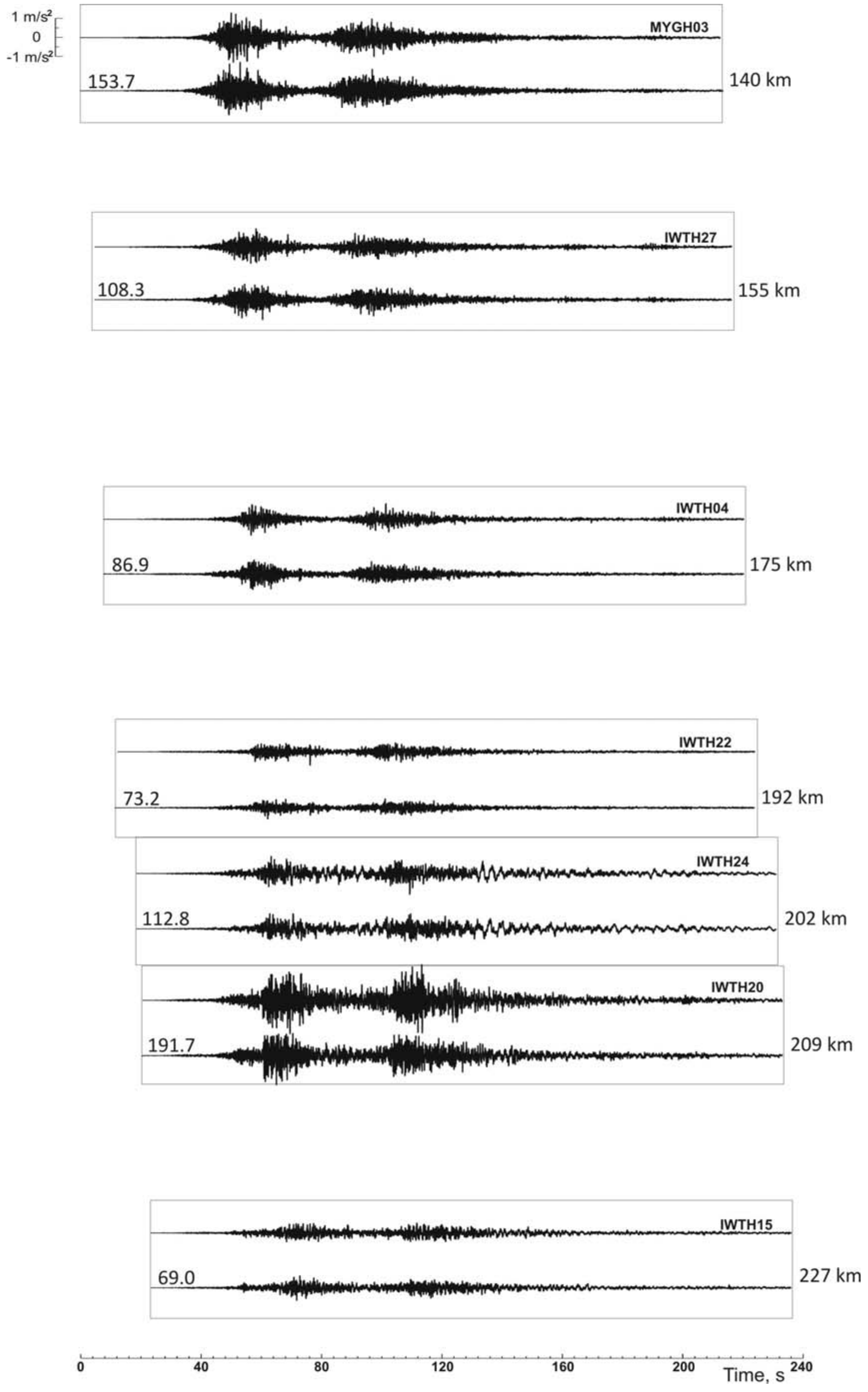

1 m/s²
0
-1 m/s²
MYGH03
153.7
140 km
IWTH27
108.3
155 km
IWTH04
86.9
175 km
IWTH22
73.2
192 km
IWTH24
112.8
202 km
IWTH20
191.7
209 km
IWTH15
69.0
227 km
0
40
80
120
160
200
240
Time, s

c

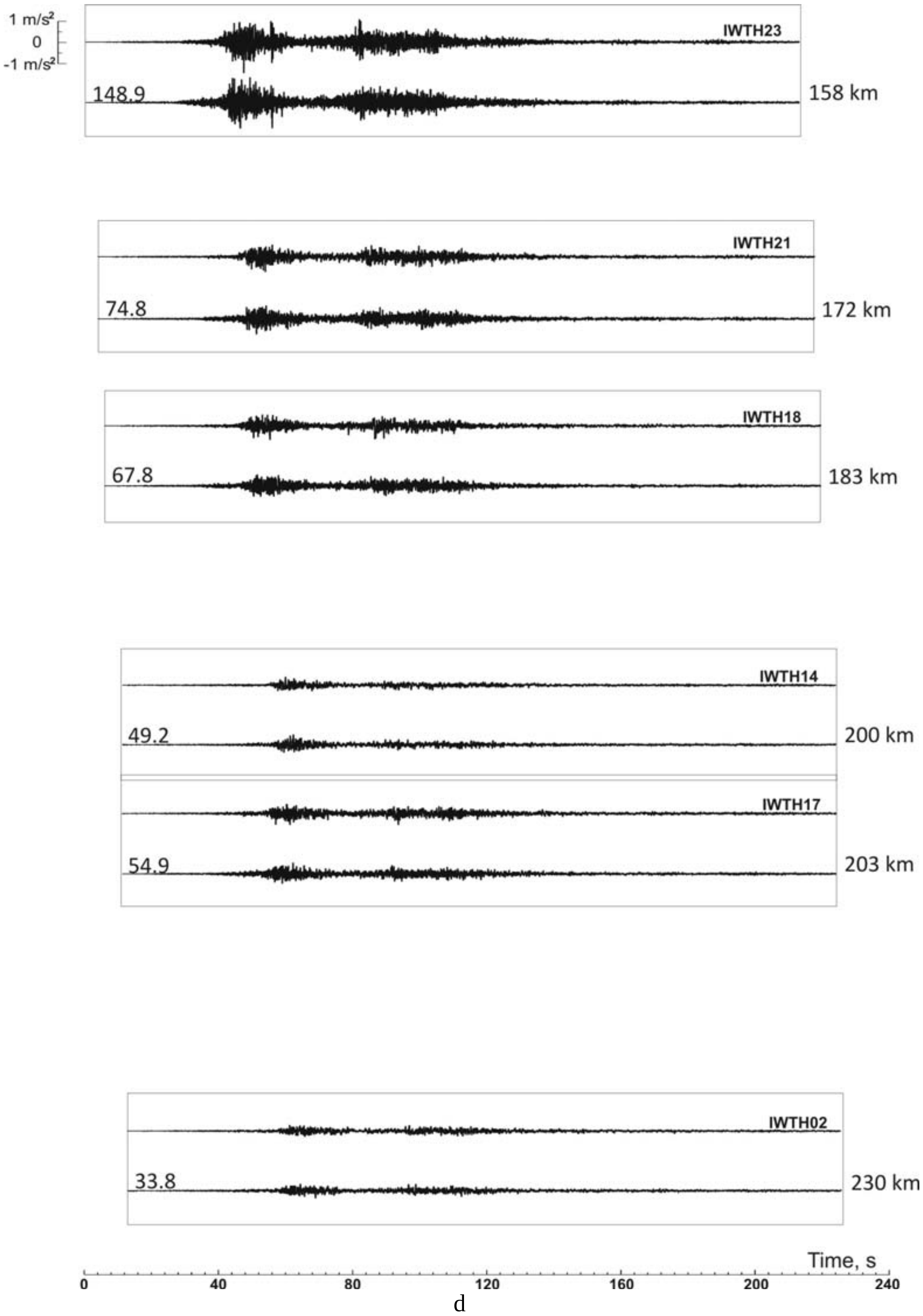

d

Figure S5. The acceleration time histories (EW and NS components, depth records) of the 2011 Tohoku earthquake at KiK-net stations located in sectors a (a), b (b), c (c), and d (d) (Fig. 1). For each station, the epicentral distances (on the right) and PGAs in cm/s^2 (on the left) are shown.
