# Supplementary material for: Features of soil behavior in the near-fault zones during the 2011 Tohoku mega-thrust earthquake Mw 9
Source: Sci Rep. 2020 May 26;10:8717. doi: 10.1038/s41598-020-65629-2 (PMC7250923; doi:10.1038/s41598-020-65629-2)
Supplement: Supplementary file 2 — Supplementary information 2. [file 41598_2020_65629_MOESM2_ESM.docx]

Supplementary Information file for the article:

**Features of soil behavior in the near-fault zones during the 2011 Tohoku mega-thrust earthquake Mw 9**

Olga V. Pavlenko

Schmidt Institute of Physics of the Earth, Russian Academy of Sciences, B. Gruzinskaya 10, Moscow 123242, Russia

e-mail: olga@ifz.ru, phone: +7 499 254 9025, mobile: +7 915 333 0260

This file is provided to contain all the necessary Figures that could not be included in the main text due to space constraints.


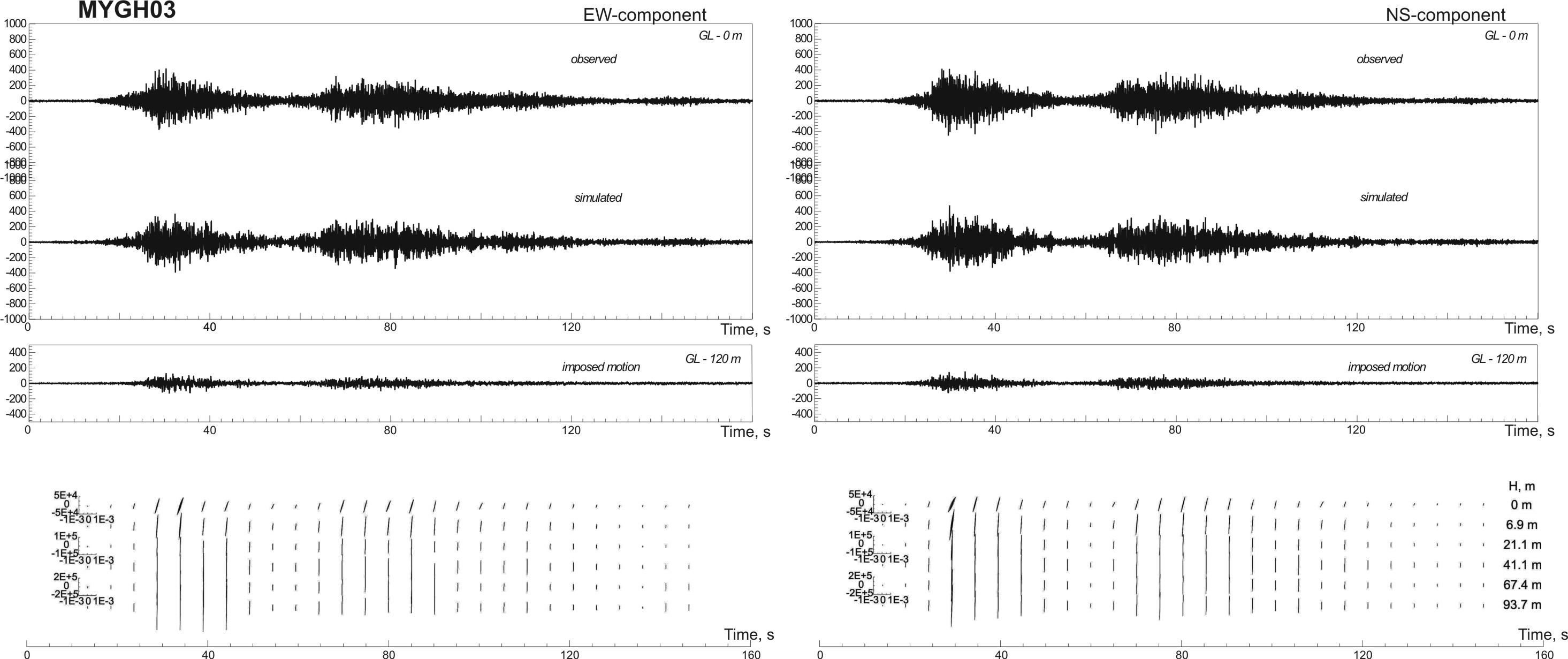


a


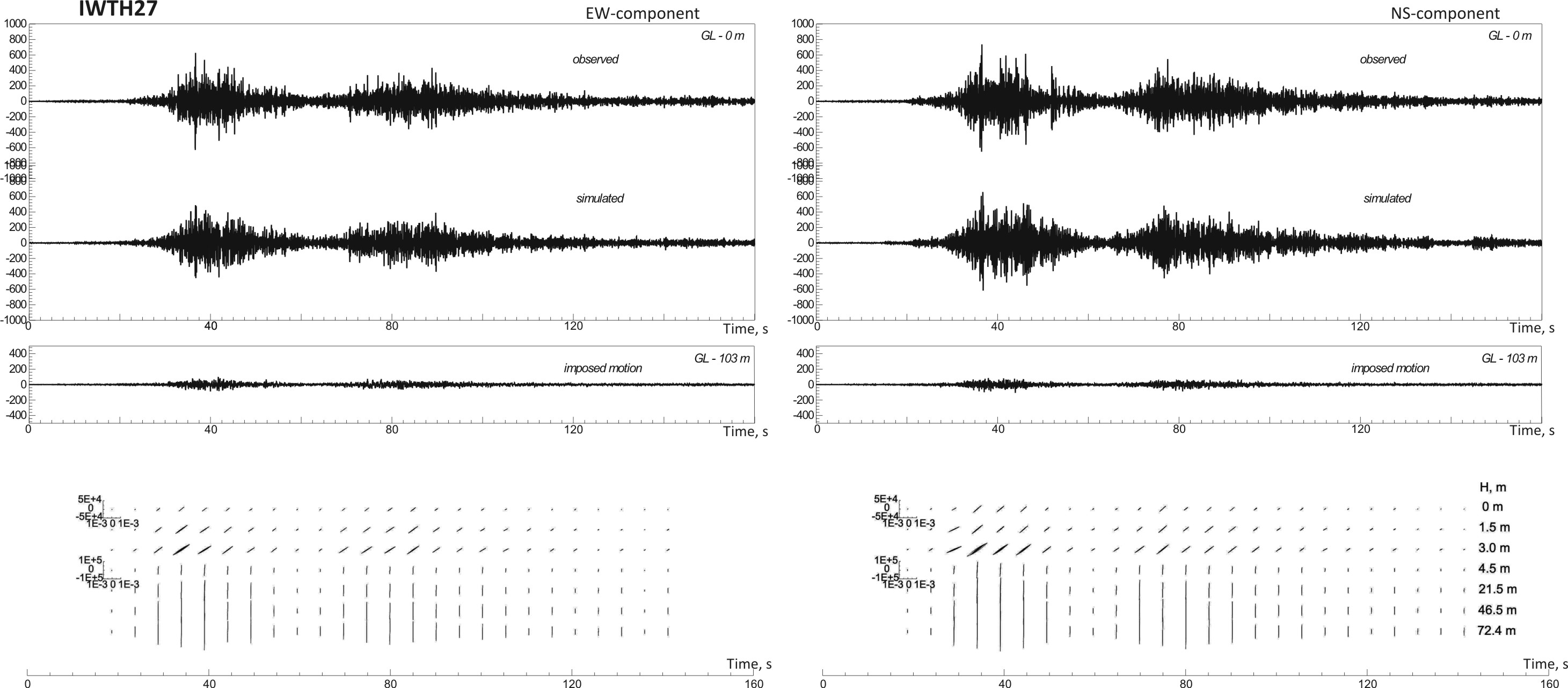


b


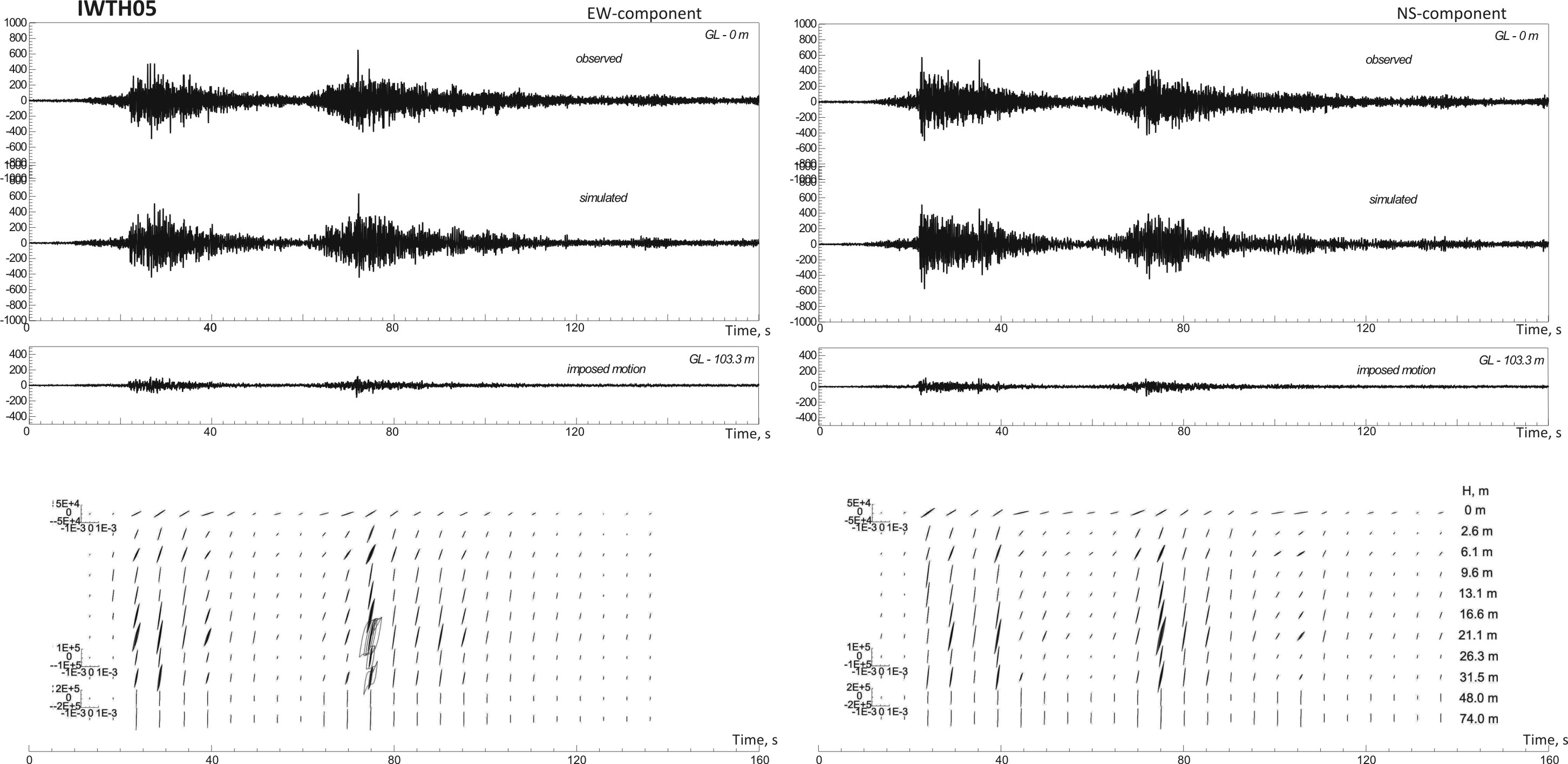


c


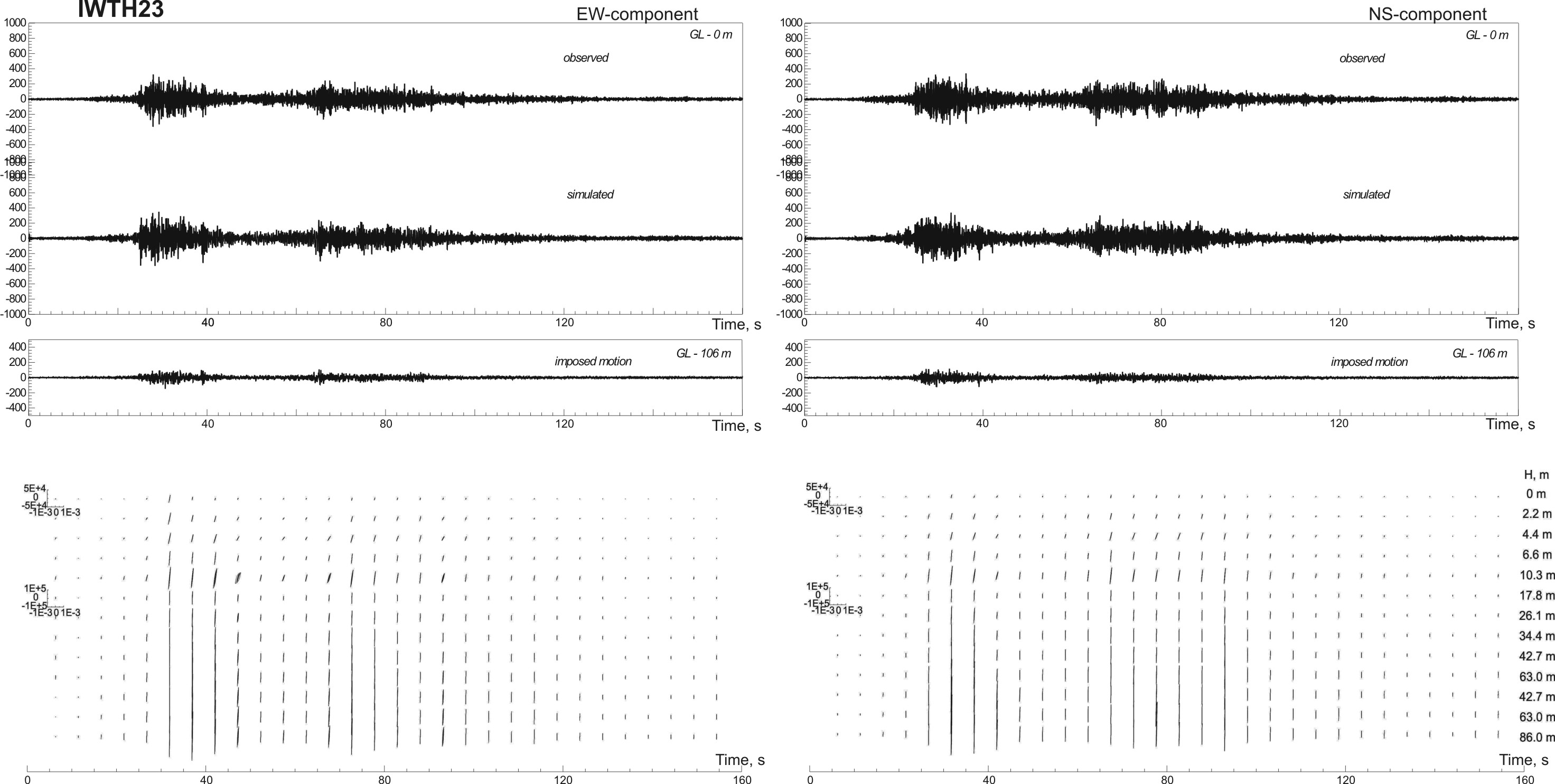


d


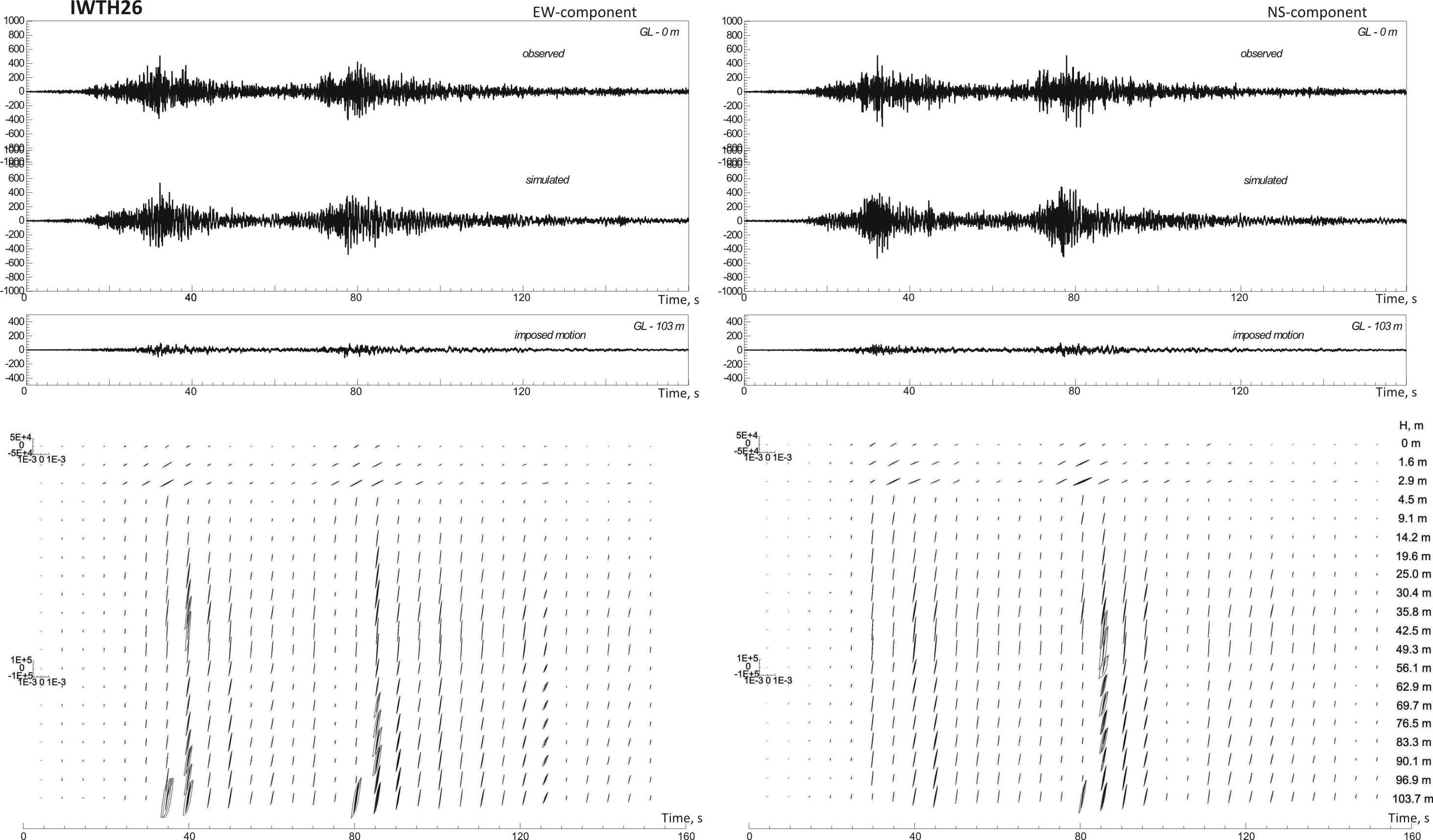


e


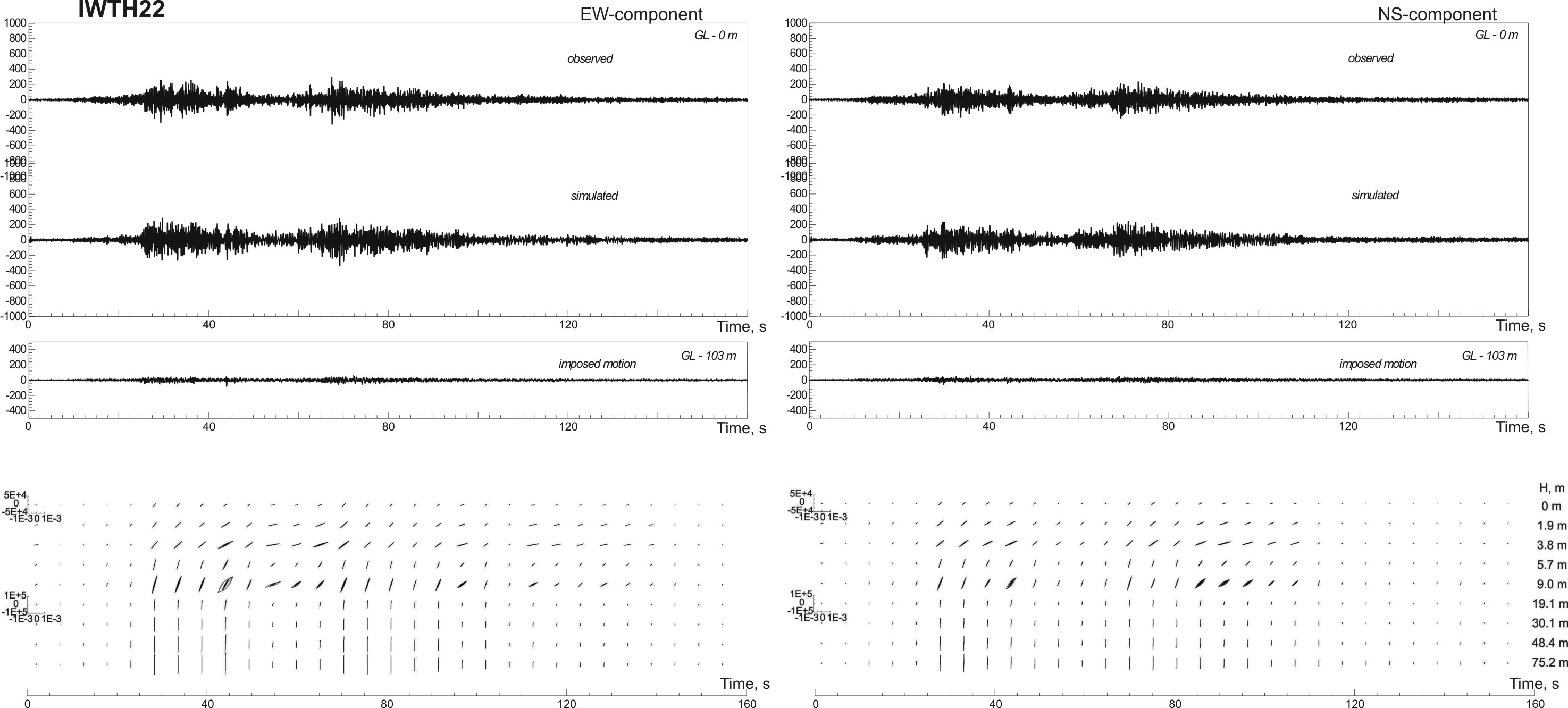


h

Figure S1. The acceleration time histories of the Tohoku earthquake, observed and simulated, and estimated stress-strain relations in soil layers, changing with time during strong motion: a - at MYGH03 station; b - at IWTH27 station; c - at IWTH05 station; d - at IWTH23 station; e - at IWTH26 station; f - at IWTH22 station. Accelerations are given in cm/s^2^, stresses are given in Pa, and strains in strains.


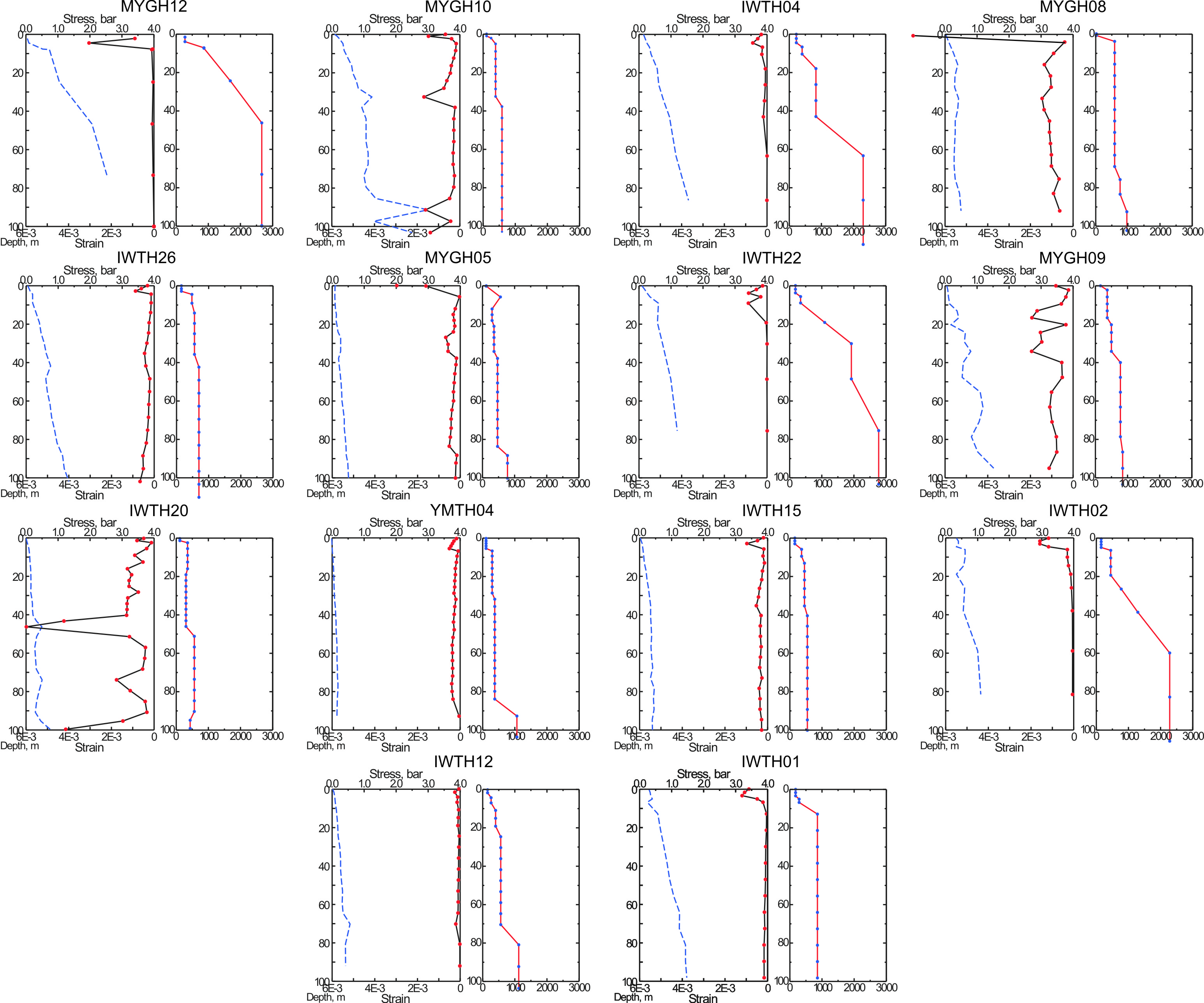
a


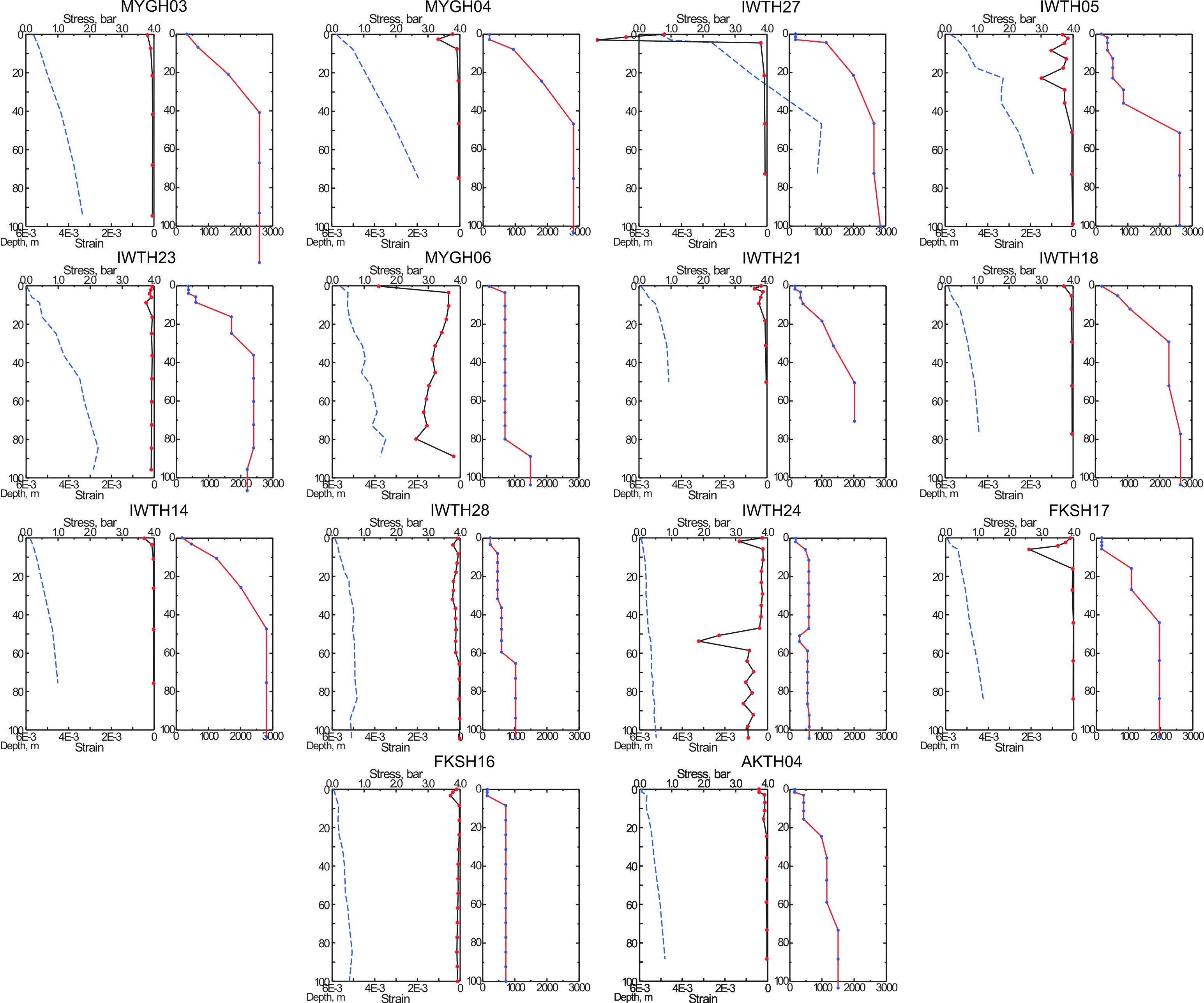
b

Figure S2. The profiling data: *Vs* velocities on the right, and the estimates of maximum stresses and strains (dash lines and solid lines, respectively, on the left) induced in the upper ~ 100 m by strong motion at the studied KiK-net sites: a – with softer subsurface soils, b – with denser subsurface soils. Stresses are given in bars, and strains in strains.


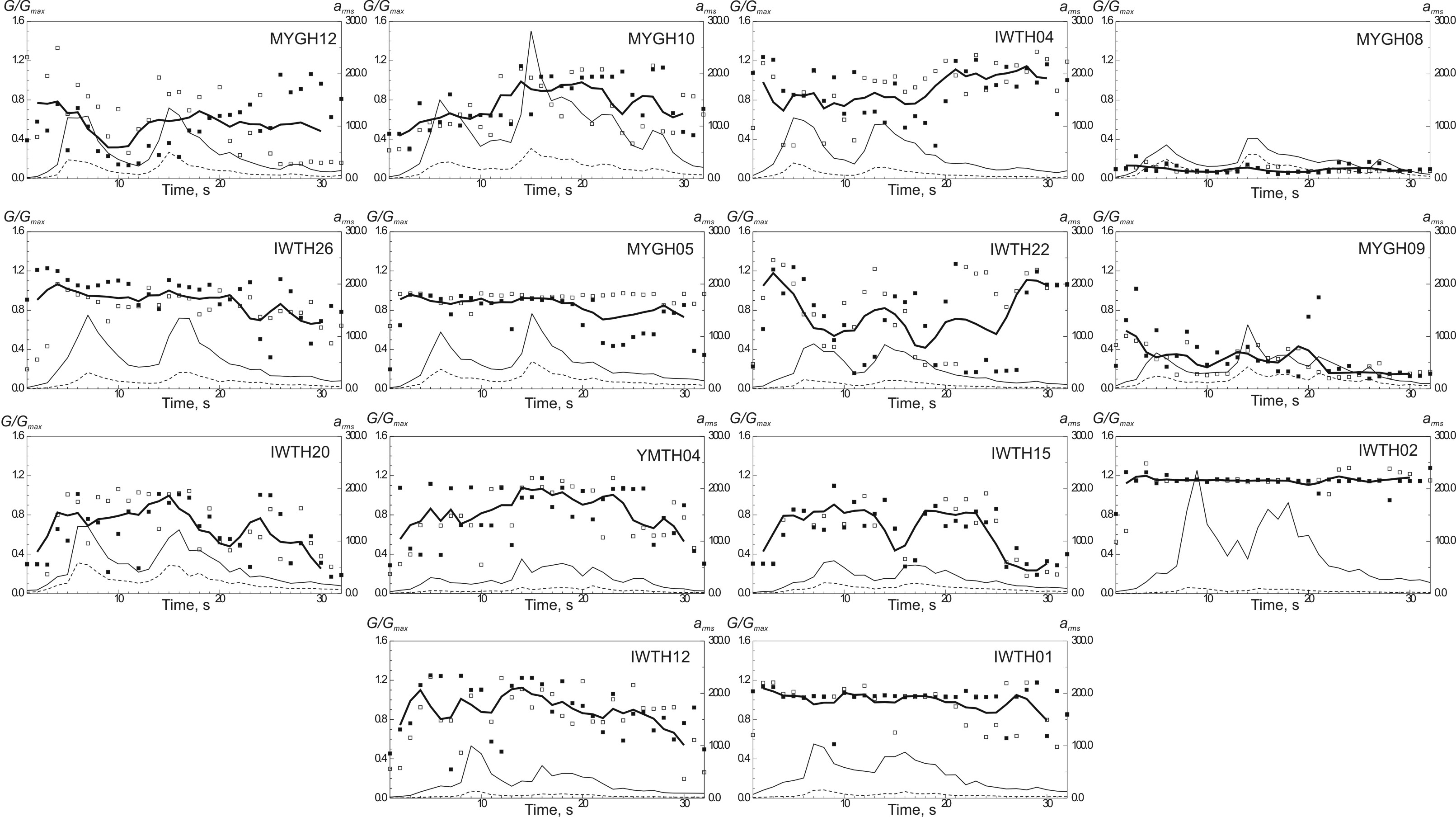
a


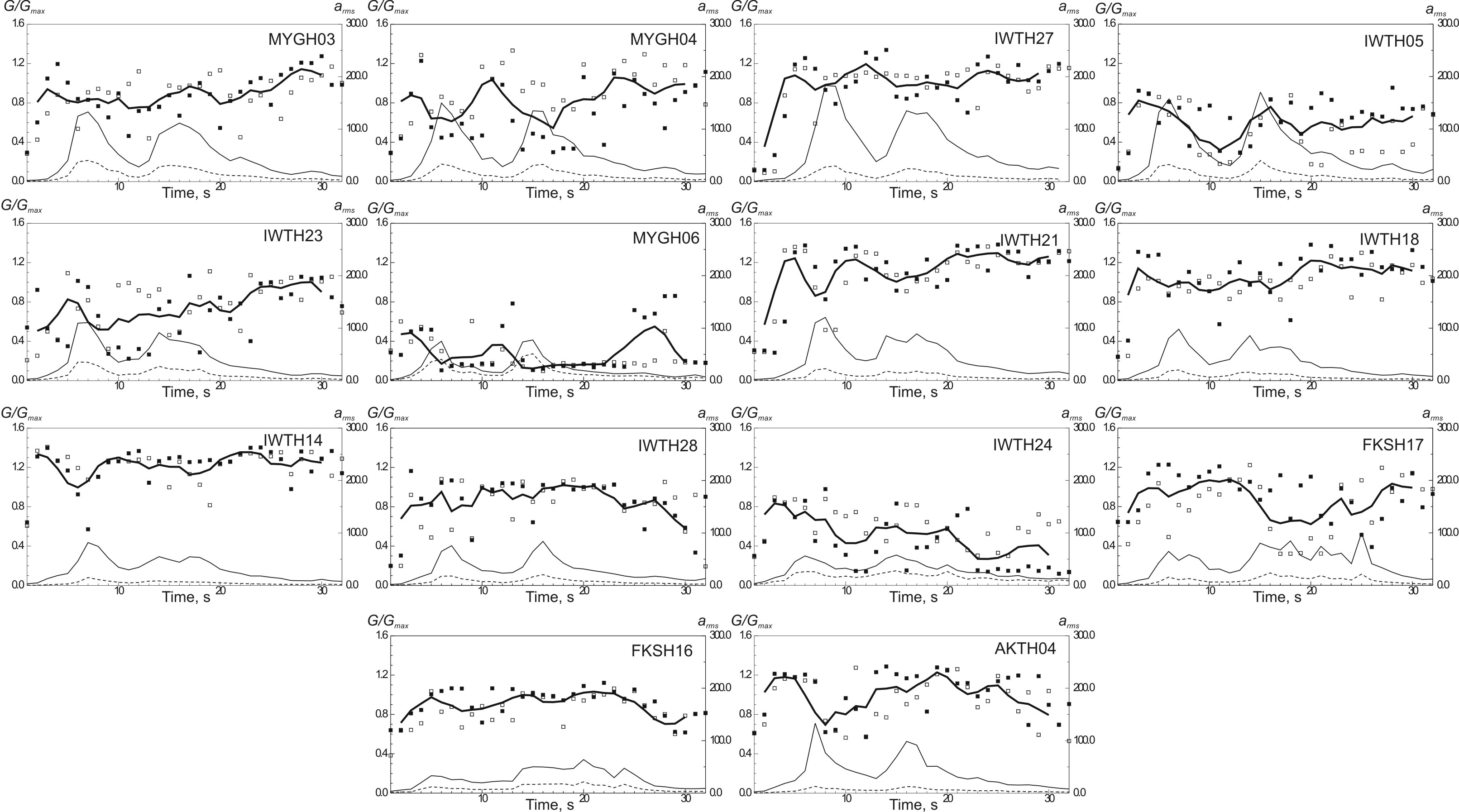


b

Figure S3. Changes of shear moduli in soil layers at the studied KiK-net sites during the Tohoku earthquake: a – at sites with softer subsurface soils, b – at sites with denser subsurface soils. Points indicate the estimated shear moduli at two horizontal components, thick lines show these values smoothed and averaged over two horizontal components. Thin lines are the intensities of motion on the surface (solid lines) and at a depth of location of the deep device (dash lines).


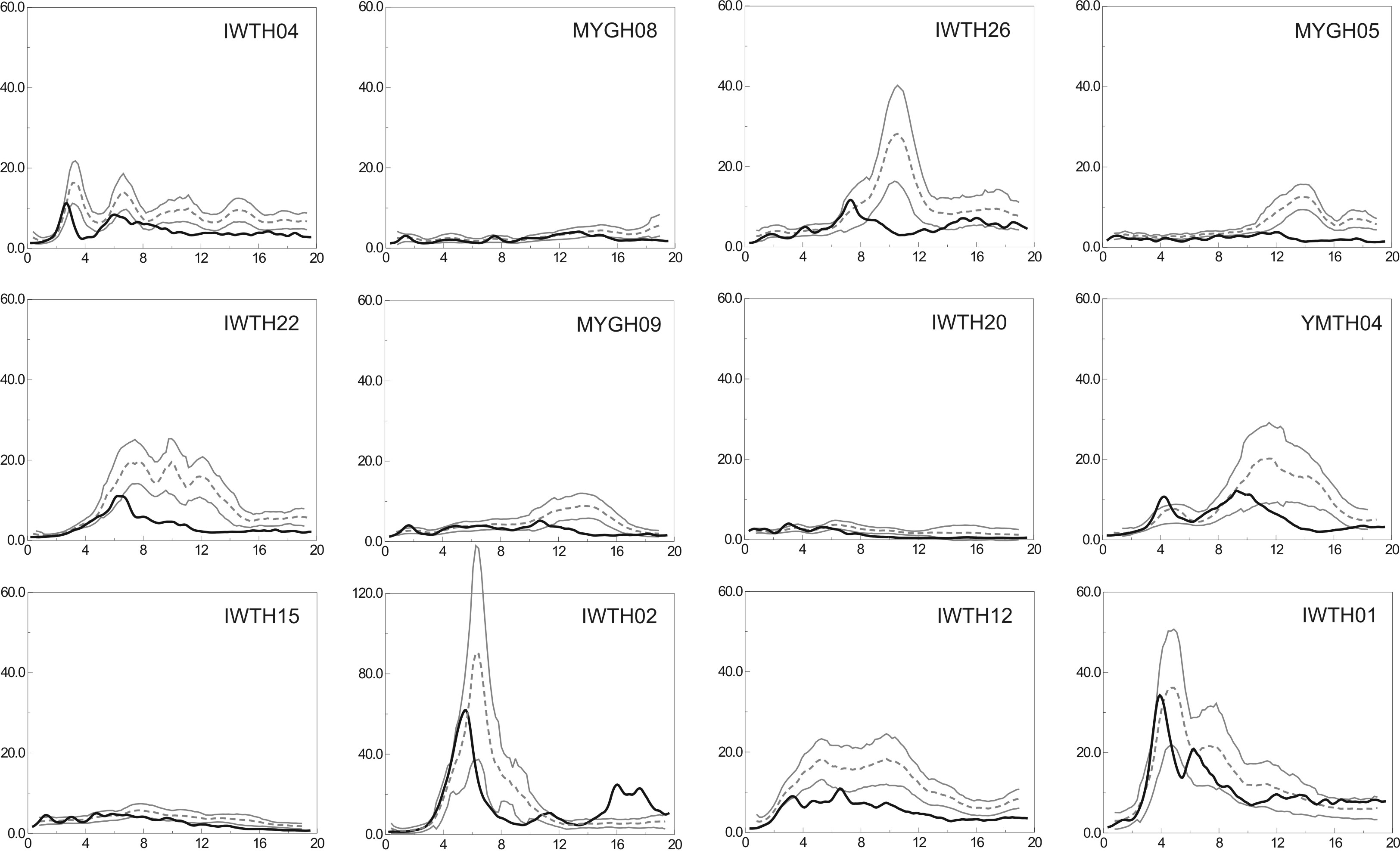


a


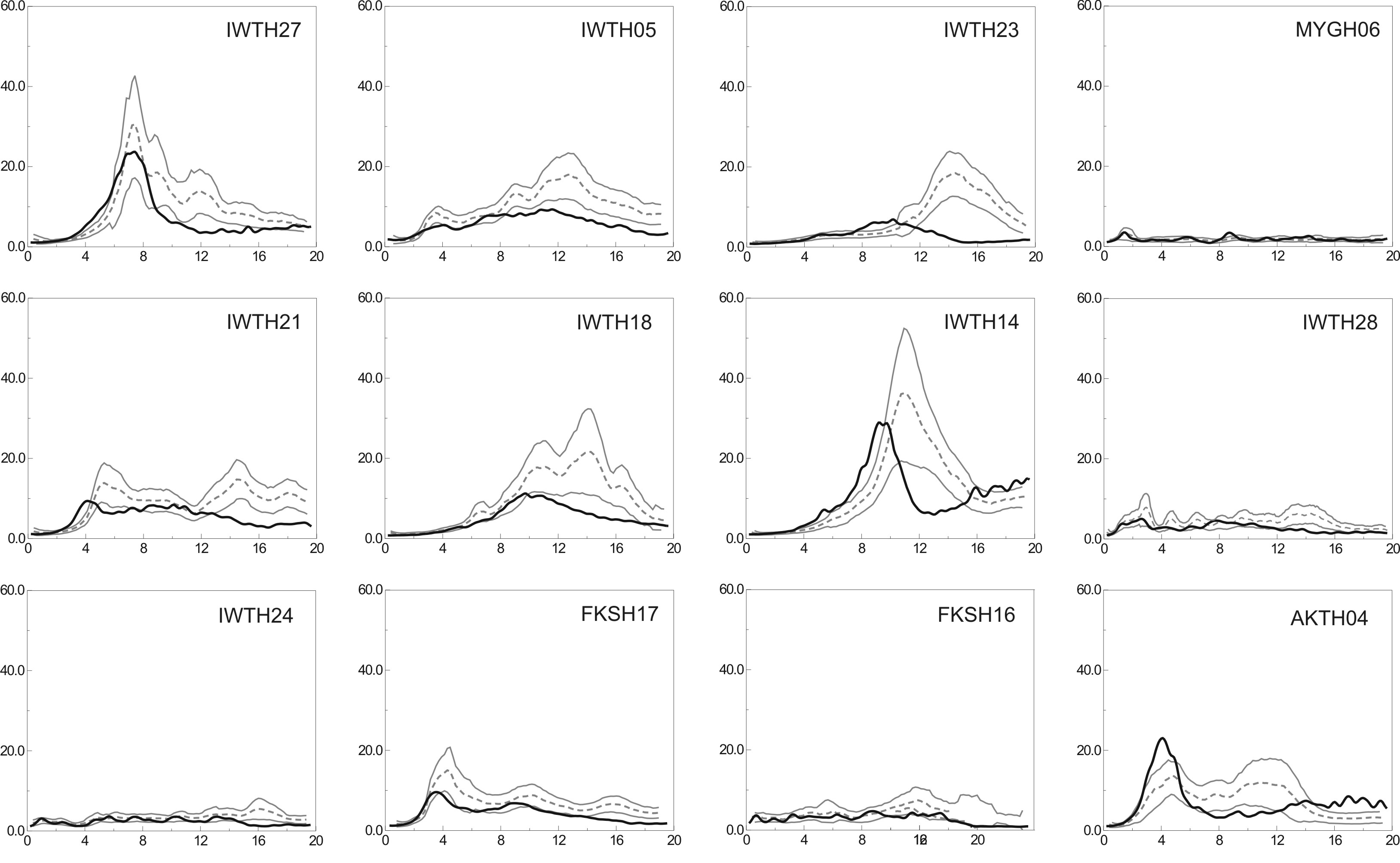


b


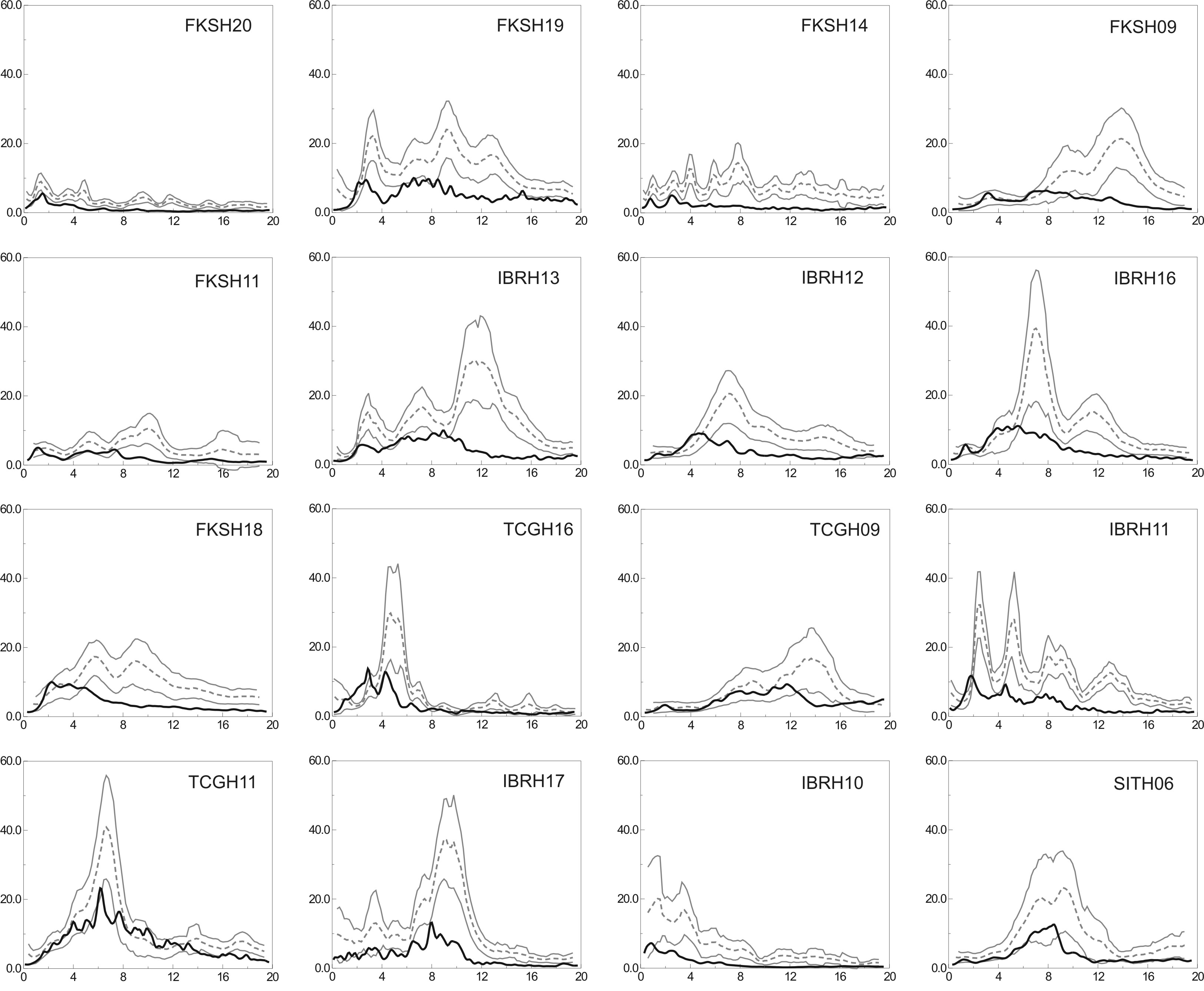


c


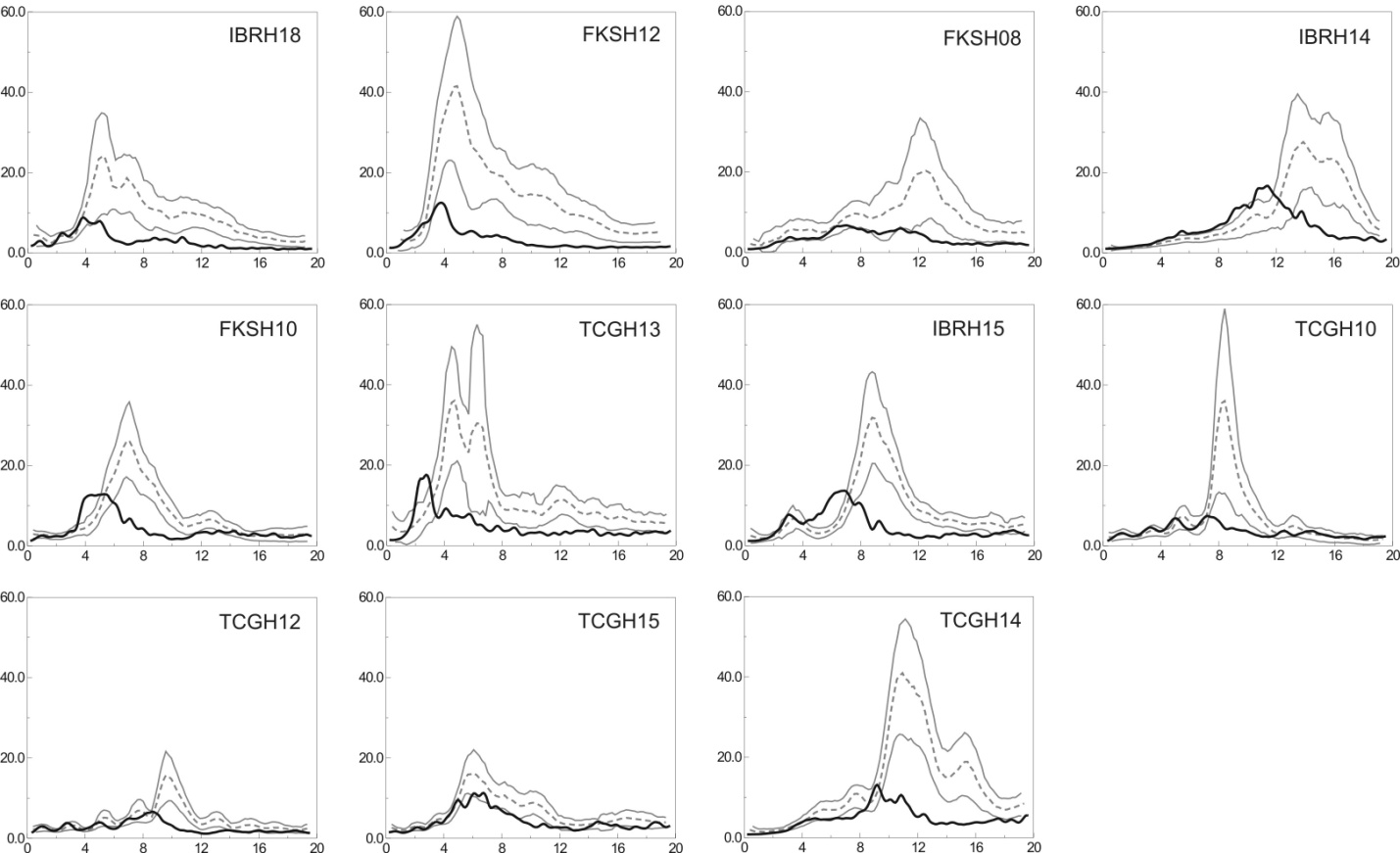


d

Figure S4. The borehole transfer functions at KiK-net sites calculated for the Tohoku earthquake *G_T_(f)* (black thick lines) and for weak motions *G_W_(f)* (thin dash lines – averages, thin solid lines – confidence limits): a – at sites with softer soils in northern Honshu; b – at sites with denser soils in northern Honshu; c – at sites with softer soils in southern Tohoku and Kanto regions; d – at sites with denser soils in southern Tohoku and Kanto regions. Abscissa is frequency.


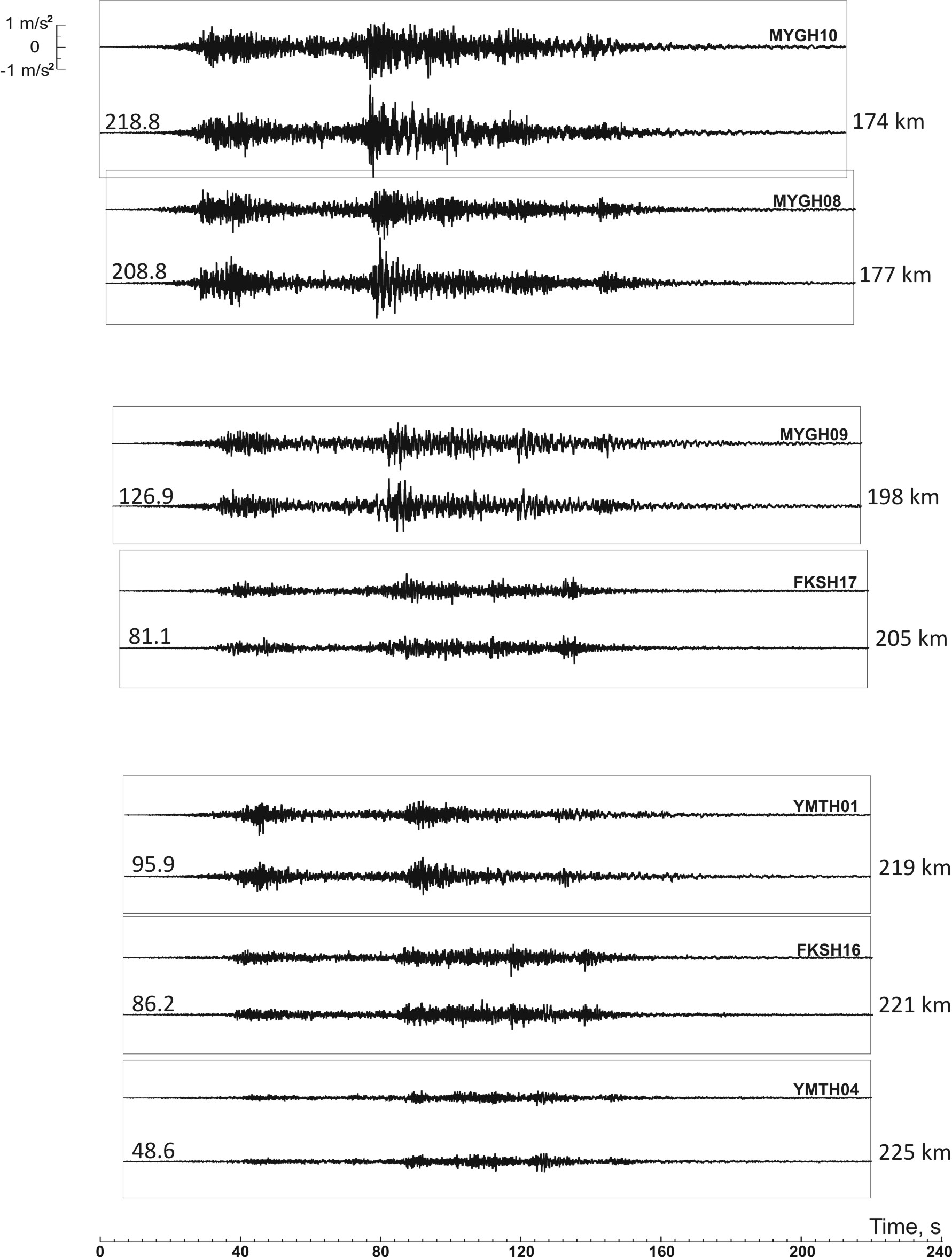


a


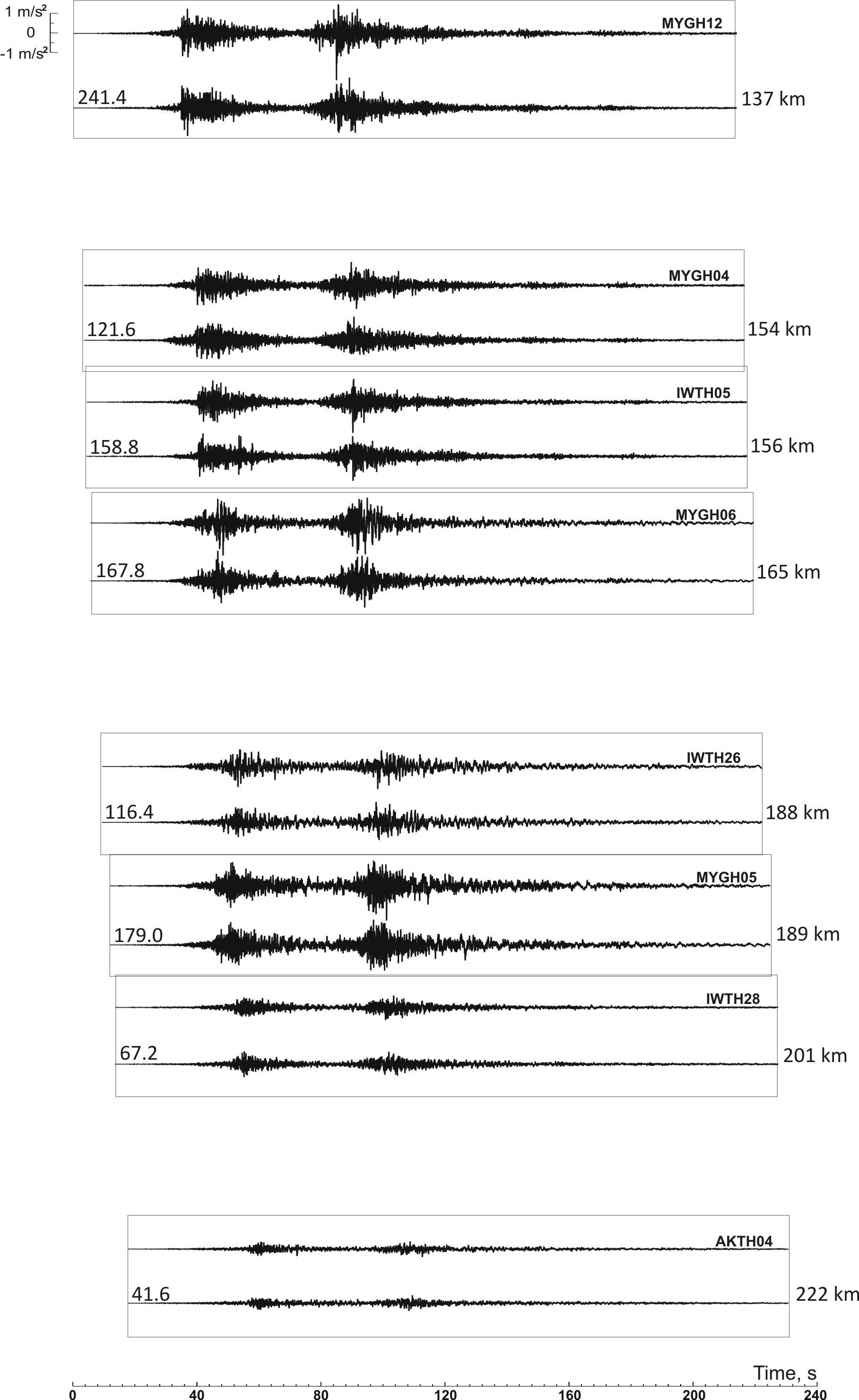


b


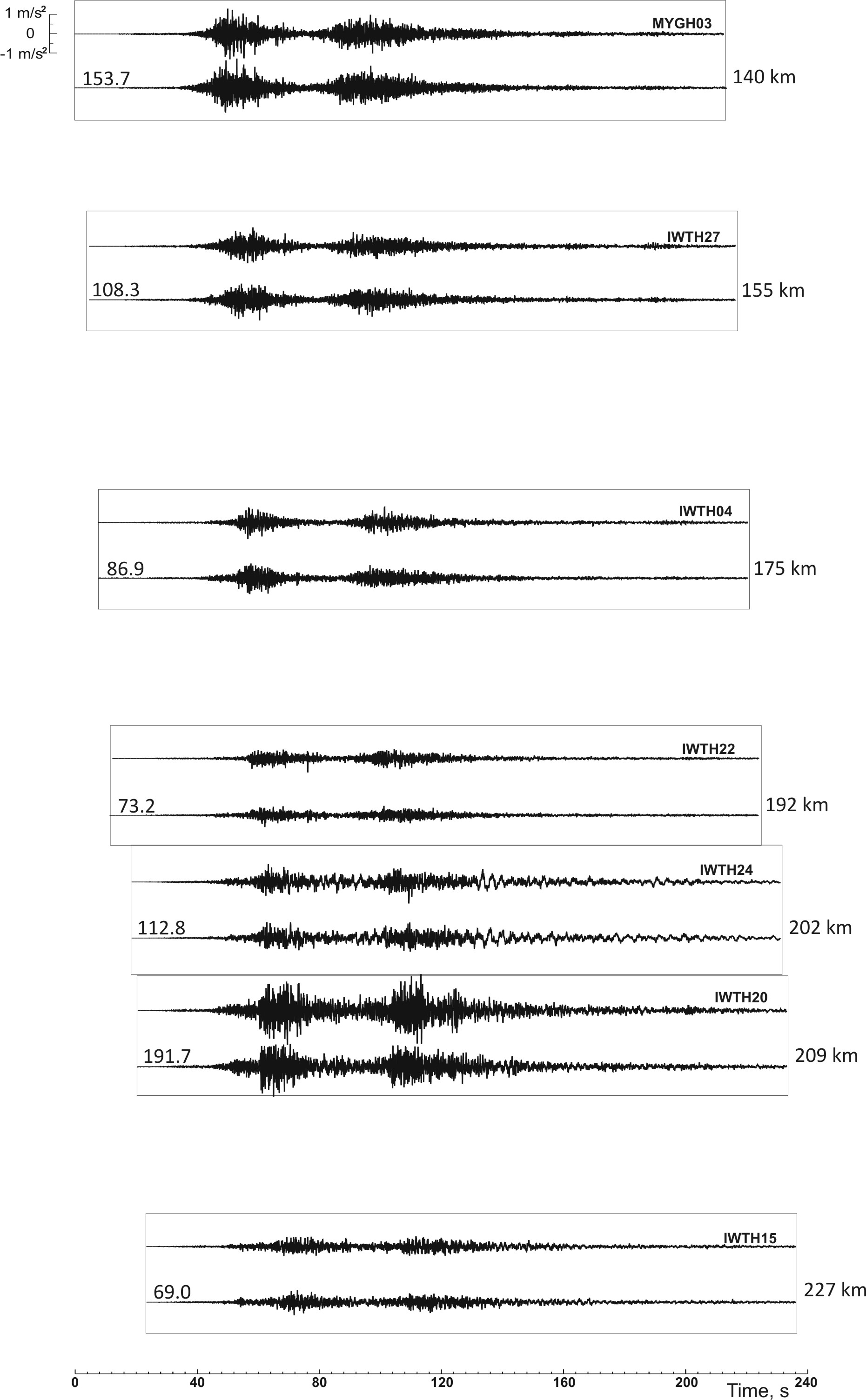


c


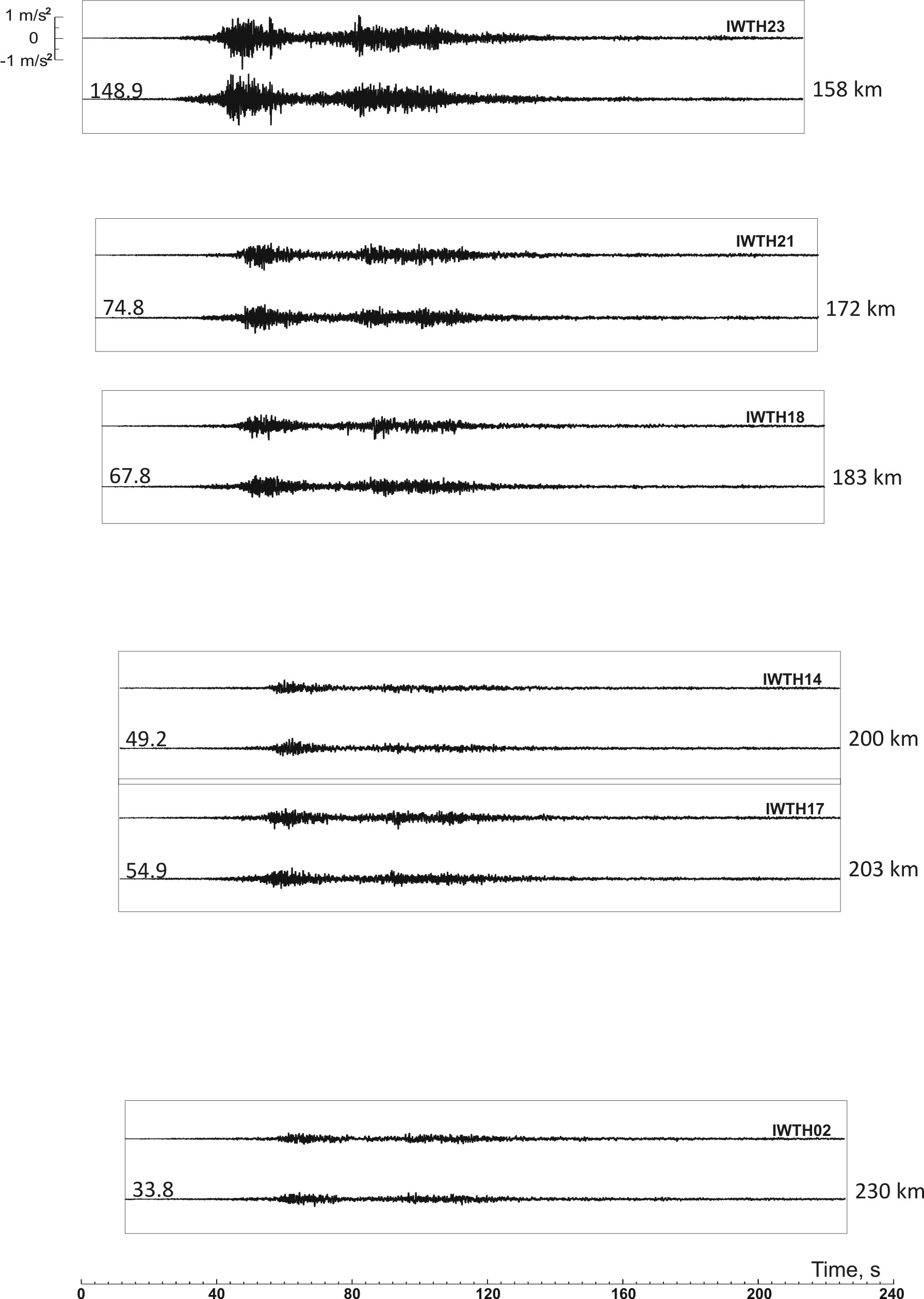


d

Figure S5. The acceleration time histories (EW and NS components, depth records) of the 2011 Tohoku earthquake at KiK-net stations located in sectors a (a), b (b), c (c), and d (d) (Fig. 1). For each station, the epicentral distances (on the right) and PGAs in cm/s^2^ (on the left) are shown.
